# Exploring Bioactive Phytomedicines for Advancing Pulmonary Infection Management: Insights and Future Prospects

**DOI:** 10.1002/ptr.8334

**Published:** 2024-10-09

**Authors:** Joyce Siaw Syuen Ho, Teh Li Ping, Keshav Raj Paudel, Tammam El Sherkawi, Gabriele De Rubis, Stewart Yeung, Philip M. Hansbro, Brian Gregory George Oliver, Dinesh Kumar Chellappan, Keng Pei Sin, Kamal Dua

**Affiliations:** ^1^ Department of Pharmaceutical Chemistry, School of Pharmacy International Medical University Kuala Lumpur Malaysia; ^2^ Centre for Inflammation, School of Life Sciences, Faculty of Science Centenary Institute and the University of Technology Sydney Sydney Australia; ^3^ Discipline of Pharmacy, Graduate School of Health University of Technology Sydney Sydney Australia; ^4^ Australian Research Centre in Complementary and Integrative Medicine, Faculty of Health University of Technology Sydney Ultimo Australia; ^5^ School of Life Science University of Technology Sydney Ultimo Australia; ^6^ Woolcock Institute of Medical Research Macquarie University Sydney Australia; ^7^ Department of Life Sciences, School of Pharmacy International Medical University Kuala Lumpur Malaysia

**Keywords:** COVID‐19, influenza, medicinal plants, pneumonia, pulmonary infection, tuberculosis

## Abstract

Pulmonary infections have a profound influence on global mortality rates. Medicinal plants offer a promising approach to address this challenge, providing nontoxic alternatives with higher levels of public acceptance and compliance, particularly in regions where access to conventional medications or diagnostic resources may be limited. Understanding the pathophysiology of viruses and bacteria enables researchers to identify biomarkers essential for triggering diseases. This knowledge allows the discovery of biological molecules capable of either preventing or alleviating symptoms associated with these infections. In this review, medicinal plants that have an effect on COVID‐19, influenza A, bacterial and viral pneumonia, and tuberculosis are discussed. Drug delivery has been briefly discussed as well. It examines the effect of bioactive constituents of these plants and synthesizes findings from in vitro, in vivo, and clinical studies conducted over the past decade. In conclusion, many medicinal plants can be used to treat pulmonary infections, but further in‐depth studies are needed as most of the current studies are only at preliminary stages. Extensive investigation and clinical studies are warranted to fully elucidate their mechanisms of action and optimize their use in clinical practice.

## Graphical abstract

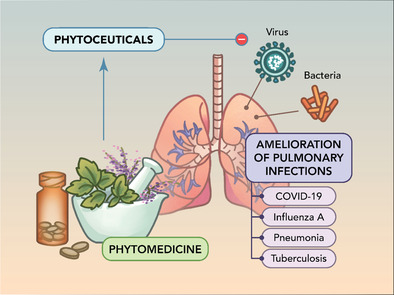

**Abbreviations**
ACE2: angiotensin‐converting enzyme 2
AIM2: absent in melanoma 2
ASC: apoptosis‐associated speck‐like protein
ATP: adenosine triphosphate
Bcl‐2/Bax: B cell lymphoma 2/Bcl‐2‐associated protein X
CBD: cannabidiol
CBG: cannabigerol
CFU: colony‐forming units
COVID‐19: coronavirus disease 2019
COX‐2: cyclooxygenase 2
CXCL‐10: C‐X‐C motif chemokine ligand 10
DPPH: 2,2‐diphenyl‐1‐picrylhydrazyl
EIF2AK2/PKR: eukaryotic translation initiation factor 2‐alpha kinase 2/protein kinase R
GC–MS: gas chromatography–mass spectrometry
GC‐Q‐TOF‐MS: gas chromatography‐quadrupole time‐of‐flight‐mass spectrometry
G‐CSF: granulocyte colony–stimulating factor
GPCR: G protein‐coupled receptor
HEK239T: human embryonic kidney cell line
HepG2: hepatoblastoma cell line
HIV‐1: human immunodeficiency virus type 1
HMGB1: high mobility group box 1 protein
HSV1: herpes simplex virus type 1
IκB‐α: nuclear factor of kappa light polypeptide gene enhancer in B cells inhibitor alpha
IAV: influenza A virus
IFN‐γ: interferon‐gamma
IKK‐α: inhibitory‐κB kinase alpha
iNOS: inducible nitric oxide synthase
IRF3/7: interferon regulatory factor 3/7
ISG‐15: interferon‐stimulated gene 15
JAK–STAT: janus kinase/signal transducers and activators of transcription
JAK2: janus kinase 2
LPS: lipopolysaccharide
MAPKs: mitogen‐activated protein kinases
MDA‐5: anti‐melanoma differentiation‐associated gene 5
MDCK: Madin–Darby canine kidney cells
mPGES‐1: microsomal prostaglandin E synthase‐1
mTOR: mammalian target of rapamycin
MX/MX‐1: myxovirus resistance/Myxoma resistance 1
NF‐κB: nuclear factor kappa B
NKT cells: natural killer T cells
NLRC4: NLR family CARD domain containing 4
NLRP3: NLR family pyrin domain containing 3
Nrf2: nuclear factor erythroid 2–related factor 2
OAS: 2′−5′‐oligoadenylate synthetase
PED virus: porcine epidemic diarrhea virus
PGE2: prostaglandin E2
PI3K/Akt: phosphoinositide 3‐kinase/protein kinase B
RIG‐1: retinoic acid‐inducible gene‐I
RT‐PCR: reverse transcription–polymerase chain reaction
STAT3: signal transducer and activator of transcription
TGFβ: transforming growth factor‐β
THC: Δ9‐tetrahydrocannabinol
TMPRSS2: transmembrane protease serine 2
TNF‐α: tumor necrosis factor alpha
TYK2: tyrosine kinase‐2

## 1 Introduction

Respiratory diseases such as acute respiratory distress syndrome (ARDS), asthma, chronic obstructive pulmonary disease (COPD), lung fibrosis, and lung cancer have exerted a profound influence on global mortality rates, claiming the lives of billions of individuals (Mehta, Dhanjal, Paudel, et al. 2020). Not to mention severe acute respiratory syndrome coronavirus 2 (SARS‐CoV‐2), widely known as COVID‐19, that was accountable for the pandemic that occurred in 2020 (Darmarajan et al. 2022). The number of reported deaths caused by COVID‐19 directly or indirectly in 2020 alone was 1,813,188, but WHO estimated that the number of excess mortalities was much higher, at a minimum of 3.0 million (WHO 2021). Furthermore, in 2019, COPD was ranked as the third most common cause of global mortality, which resulted in 3.23 million deaths (WHO 2023b). As for influenza, which can lead to viral pneumonia, was ranked 12th in the cause of deaths, with 47,052 fatalities in the United States in 2022 (CDC 2024). On the other hand, tuberculosis (TB) had a decreasing trend in mortality rates globally until 2020, when progress was reversed due to disruptions caused by COVID‐19. However, there was a decrease in 2022, resulting in an overall reduction in mortality of 19% from 2015 to 2022, which is still far from the WHO End TB Strategy milestone of a 75% reduction by 2025 (WHO 2023a). Hence, it is clear that respiratory diseases can be fatal, especially among infants, young children, and the elderly, whose immune systems are either underdeveloped or impaired. The socioeconomic impact of these disorders has significantly hampered the well‐being of affected individuals, with predictions indicating a substantial rise in mortality due to inflammatory respiratory diseases in the foreseeable future (Mehta, Sharma, et al. 2020).

Different approaches to prevention and managing diseases have been put into practice in both developing and developed nations with the aim of improving the well‐being of affected individuals. However, a notable challenge lies in the undertreatment of these conditions, often attributed to factors such as the unavailability of vaccines/medications, diagnostic limitations, and the lack of awareness or attention from patients and their families (Mehta, Sharma, et al. 2020). For countless millennia, medicinal plants have been utilized in traditional medicine across diverse nations on a global scale (Bani Saeid et al. 2023; Devkota et al. 2022; Kokkinis et al. 2024; Paudel, De Rubis, et al. 2022; Prasher et al. 2020). The practical insights into the positive impacts of plants have been passed down through generations. It has been indicated in history that medicinal plants have been involved in treating various pandemics prior to the introduction of significant vaccines (Pranskuniene et al. 2022). However, there is still much potential to expand the use of various medicinal plants as many species remain unstudied or require further in‐depth research (Marrelli 2021).

The usage of herbs brings many advantages as compared to modern medicine, ranging from cost‐effectiveness and enhanced global accessibility to reduced adverse events, greater public acceptance, and a diminished reliance on conventional medications and hospitalization (Hajimonfarednejad et al. 2023; Nyagumbo et al. 2022). Nevertheless, there are also various concerns surrounding the use of medicinal plants, including their efficacy, toxicity, dosage, and long‐term effects. Medicinal plants are usually deemed safe to consume in the public eye, and any potential health adverse effects that may arise from prolonged use are often easily overlooked. Some substances present in plants are toxic, such as phenols and carvone, which can result in various health issues if taken in excess (Pranskuniene et al. 2022).

### 1.1 Medicinal Plants in Pulmonary Infections

Diverse traditional medical systems such as Unani, Persian, Chinese, Indian, and other alternative systems have addressed the issue of respiratory infections and their corresponding therapeutic interventions. Unani medicine can be traced back to ancient Greece, rooted in the teachings of Hippocrates. An example of Unani medicine used to combat pulmonary infections is Tiryaq Arba, which contains the berry of *Laurus nobilis*, oleogum resin of *Commiphora myrrha*, and roots of *Gentiana lutea* and *Aristolochia longa*. These constituents have been tested and proven to have antiviral activity, which can combat against multiple respiratory viruses (Ansari et al. 2020; Khan, Bashir, and Akhtar 2020). As for Persian medicine (PM), it stands as an ancient medical paradigm offering a plethora of approaches for disease management and complication mitigation. Notably, PM references document numerous remedies for a wide range of treatments for different respiratory ailments, encompassing conditions such as asthma and pneumonia (Hajimonfarednejad et al. 2023). Plants such as *Alpinia galanga* (galangal), *Crocus sativus* (saffron), and *Glycyrrhiza* spp. (licorice) are used to treat various pulmonary diseases in PM (Bahramsoltani and Rahimi 2020).

**FIGURE 1:**
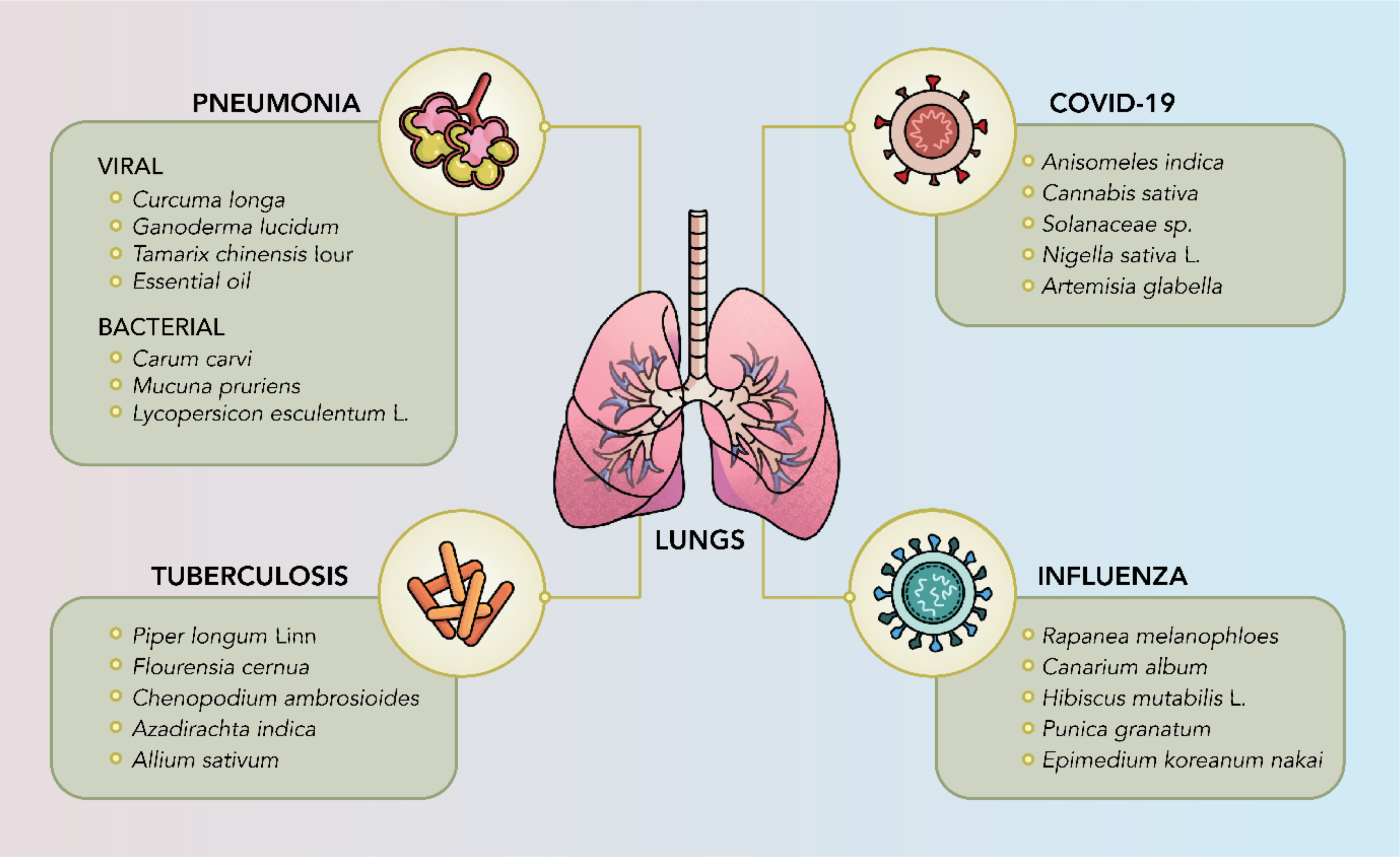
Pulmonary infections and examples of medicinal plants that can treat them.

On the other hand, traditional Chinese medicine (TCM) employs many approaches to address health issues, including psychological, physical and herbal treatments (Traditional Chinese Medicine 2019). Medicinal plants such as *Ephedra*, *Scutellaria baicalensis*, *Rheum officinale*, and more have been utilized not only for addressing respiratory infections but also for treating various other ailments. Similarly, *Glycyrrhiza* is also widely involved in TCM. Since Japanese Kampo medicine was adapted from TCM, the medications used are similar. For example, *Ephedrae Herba*, also known as Maoto, is used to treat influenza in Japan. Another medication, Shahakusan, composed of four medicinal plants, is used to treat pneumonia in children (Hokari, Nagai, and Yamada 2012). As for the western region, medicinal plants that are more native to the regions are used, such as *Nigella sativa*, *Aesculus hippocastanum* to name a few (Md. M. Rahman et al. 2022; Tang et al. 2023). Figure 1 shows a summary of medicinal plants mentioned in this article that can treat various pulmonary infections.

Other than the systems mentioned above, many other plants are also involved in treating pulmonary infections all over the world. For instance, the genus *Litsea*, a diverse group of evergreen trees and shrubs with around 400 species, can be found in tropical and subtropical regions and has been used in traditional medicine worldwide for treating various diseases, including influenza (Y.‐S. Wang et al. 2016). Another plant, *Mesosphaerum suaveolens*, is traditionally used for treating various diseases, with its leaves employed in the treatment of flu in Brazil and pneumonia in India (Almeida‐Bezerra et al. 2022). In traditional European medicine, plants such as *Scrophularia scorodonia* and *Sambucus nigra* are involved in treating pulmonary infections (Md. M. Rahman et al. 2022). *S*. *scorodonia* contains saikosaponins, which were isolated and tested to have antiviral and anti‐inflammatory properties, making it active against a range of viruses, including influenza virus (Cheng et al. 2006). Similarly, *S*. *nigra* flowers and berries, which contain flavonoids like rutin, have been traditionally used to alleviate fevers, coughing, nasal congestion, and also act as an antiviral against influenza (Santin et al. 2022). On the other hand, in traditional African medicine, *Vepris* plants (Rutaceae family) play a significant role in primary health care, with *Vepris simplicifolia* and *Vepris trichocarpa* used to treat pneumonia and *Vepris lanceolata* and *Vepris uguenensis* employed to treat influenza (Ombito, Chi, and Wansi 2021). In Southern Africa, up to 257 plants were identified as being used in various traditional healing systems within the region to treat viral pulmonary infections, including plants from families such as Apiaceae, Celastraceae, Lamiaceae, and more (Cock and Van Vuuren 2020).

In the Ayurvedic system, *Withania somnifera*, widely known as Ashwagandha, has a wide range of pharmacological activities, which includes anti‐inflammatory, antioxidant, and immunomodulatory effects (Singh et al. 2011). It had been proposed as a possible treatment for COVID‐19 (Saggam et al. 2021). In Siddha medicine, which originated in Southern India, medicinal plants such as *Glycyrrhiza glabra* and *Andrographis paniculata* have been used to treat COVID‐19 (Prakash et al. 2021). Additionally, another poly‐herbal formulation, *Kabasura Kudineer*, was used to treat H1N1, and in silico studies have shown its potential in treating COVID‐19 (Kiran et al. 2020; Prakash et al. 2021). In traditional Korean medicine, the two most frequently prescribed medications to treat common cold are Socheongnyongtang and Samso‐eum. Specifically, Socheongnyongtang is a mixture of eight herbs, while Samso‐eum is extracted from Sappan Lignum obtained through boiling (Kim et al. 2021). Meanwhile, in homeopathic medicine, medicinal plants such as *Bryonia alba* is used for pneumonia, and *Eupatorium perfoliatum* is used to treat common cold and flu (Hensel et al. 2011; V. Joshi and Joshi 2013). These diverse approaches highlight the global reliance on medicinal plants for managing pulmonary infections.

### 1.2 Pathophysiology of Pulmonary Infections

Both medical‐induced suppression of the immune system and those stemming from HIV‐1 have given rise to opportunistic infections. These infections pose diagnostic complexities for pathologists (Kradin and Mark 2018). Generally, the vibrissae in the nasal cavity and the mucociliary layer lining the respiratory system function by trapping particles and microorganisms. They guide these particles upward toward the pharynx for expulsion through expectoration or ingestion. Within the lung tissue, macrophages perform phagocytosis, targeting small particles and microorganisms. Additionally, immunoglobulin A in mucosal secretions strengthens the humoral immune response. If humoral immunity or nonimmune defense systems are compromised, susceptibility to pyogenic bacterial infections rises (Brink 2012).

Pulmonary infections can arise due to various factors such as alterations in lung structure, reduced ability to clear mucus, and weakened immune responses (Kradin and Mark 2018). Various factors, such as a weakened cough reflex and impaired bactericidal activity of alveolar macrophages due to factors like smoking, alcohol, and oxygen‐related issues, collectively elevate the risk of respiratory tract infections (Brink 2012). Besides, viral respiratory infections happen when a virus enters respiratory cells via inhaled particles or direct contact with the nose or eyes. These viruses are released by infected people through sneezing, coughing, or even breathing. When someone comes into contact with a contaminated surface and then touches their face, transmission takes place (Subbarao and Mahanty 2020).

**FIGURE 2:**
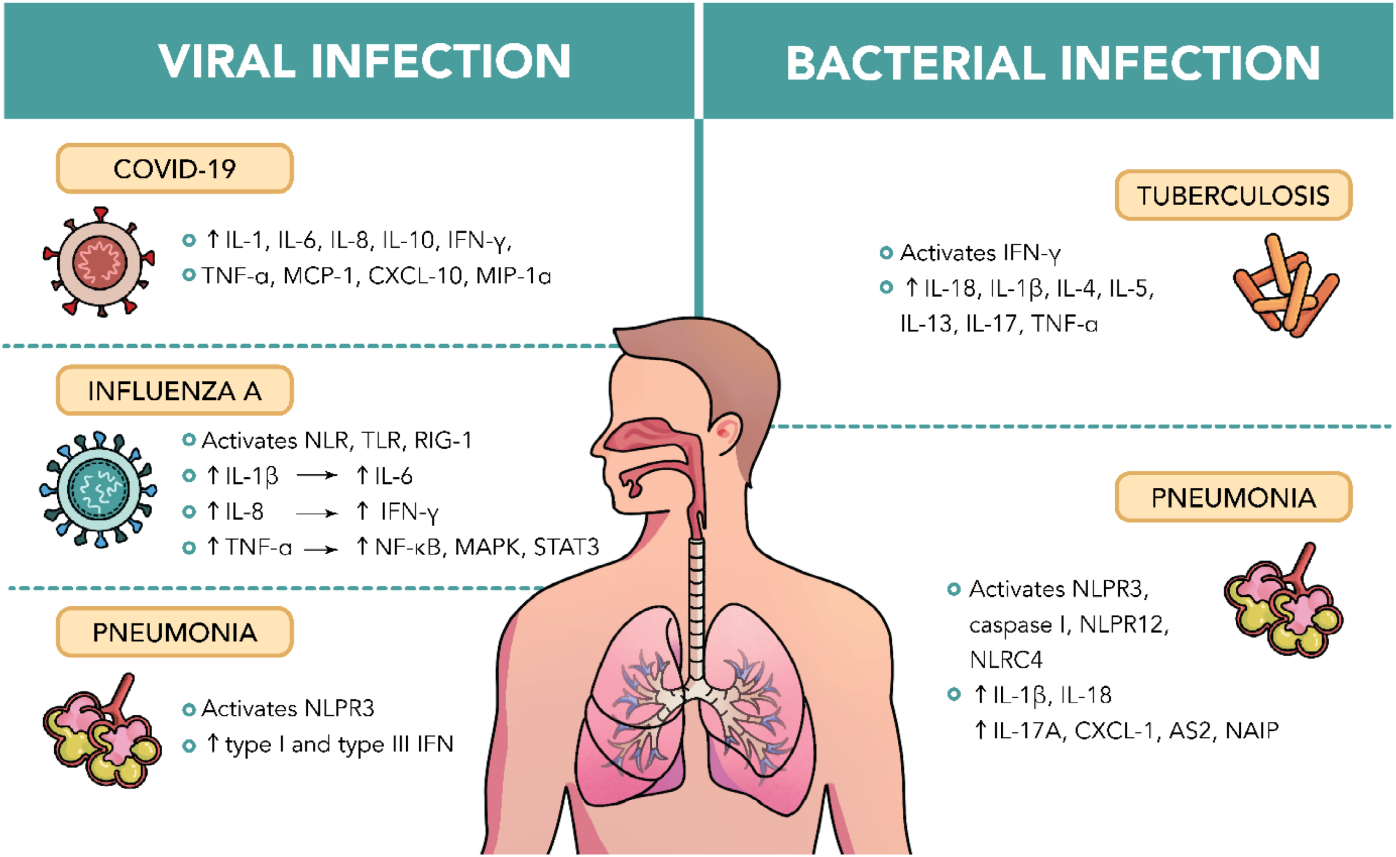
Inflammasomes that are activated and upregulated in various pulmonary infections.

Hence, a comprehensive understanding of pathophysiology has empowered clinicians to pinpoint specific risk‐associated biomarkers, allowing for the strategic combination of these markers to precisely target interventions as needed (Howes, Mcguire, and Kapur 2008). For instance, SARS‐CoV‐2, which has a diameter ranging from 60 to 140 nm and unique spikes measuring 9 to 12 nm, exhibits a distinctive appearance reminiscent of a solar corona. It can adapt to new hosts through genetic recombination and variation (Wiersinga et al. 2020). Over the past 3 years, there has been an evolution of the virus, with certain strains identified as variants of concern, presenting heightened risks to global public health. These variations encompass Beta (found in South Africa in May 2020), Gamma (found in Brazil in November 2020), Delta (found in India in October 2020), and Omicron (found in several countries in November 2021). The first samples of Alpha were found in the United Kingdom in September 2020 (S. Shen et al. 2022). SARS‐CoV‐2 specifically targets the nasal epithelial cells in the upper respiratory tract when breathed, using ACE‐2 as the main entry receptor. After the initial binding, the virus replicates locally and spreads to infect cells lining the airways. This early phase lasts a few days, which prompts a restrained immune response. Even with low viral levels during this period, individuals are highly infectious, detectable through nasal swab testing (Parasher 2021). Influenza virus, another respiratory ailment that can result in higher mortality diseases like pneumonia or ARDS, primarily replicates in the respiratory epithelium. The effective cleavage of the hemagglutinin (HA) molecule in this area generates infectious viral particles, initiating a pathological process focused on lung inflammation and compromise due to the direct viral infection of the respiratory epithelium (Kalil and Thomas 2019). Figure 2 shows inflammasomes that are activated and upregulated when infection occurs in various pulmonary infections.

## 2 Drug Delivery

Drug delivery systems encompass a range of technologies dedicated to transporting drugs within the body (Imran, Jha, Hasan, Shrestha, et al. 2022; Mehta et al. 2020; Prasher et al. 2021). It is designed to enhance the effectiveness and safety of the drug by controlling the body's rate, location, and time of release (De Rubis et al. 2023; Mehta, Dhanjal, Satija, et al. 2020; Mehta et al. 2021). There are various routes where a drug can enter the human body, which is picked based on the intended effect and the nature of the disease (Jain 2020). However, traditional drug delivery systems face challenges with limited bioavailability, unstable plasma drug concentrations, and unsatisfactory sustained release (Adepu and Ramakrishna 2021). Limitations in the bioavailability of active components from natural products involve various factors, which include low solubility of active ingredients, susceptibility to instability caused by gastric and colonic acidity, susceptibility to gut microflora affecting metabolism, limited absorption through the intestinal wall, inefficiencies in active efflux mechanisms, and the impact of first‐pass metabolic effects (H. S. Rahman et al. 2020). Hence, significant strides in drug delivery have been made over recent decades, with ongoing expectations for even more significant innovations. Ongoing developments in this field seek to improve drug delivery by precisely targeting specific areas while minimizing side effects, by using drug delivery vehicles such as micelles, liposomes, or nanoparticles (NPs) (Kaur et al. 2022, 2023; Khursheed, Dua, et al. 2022; Khursheed, Paudel, et al. 2022; NIBIB 2022; Wadhwa et al. 2021). These technological discoveries have revolutionized drug delivery by enabling the incorporation of substances with varying properties into the same formulation (Paudel et al. 2020, 2021). They can even alter the properties and behavior of a substance within a biological environment (Bonifácio et al. 2014).

In addition to facilitating controlled drug release rates, methods such as sustained delivery and targeted administration have garnered considerable interest and active pursuit. Analogous advancements involving various compounds have yielded a multitude of novel apparatuses, principles, and methodologies collectively referred to as controlled‐release technology (CRT). A diverse range of available technologies includes aerosol sprays for nasal and buccal administration, transdermal and transmucosal controlled‐release methods, medication‐infused lozenges, oral soft gel formulations, encapsulated cellular structures, iontophoretic devices tailored for transdermal drug delivery, and a variety of programmable implanted mechanisms for dispensing drugs (Tiwari et al. 2012). In advancements of drug delivery systems, effective delivery techniques have utilized formulations based on lipid and polymeric NPs, such as liposomes and cyclodextrins (Gupta et al. 2022; Harish et al. 2023; Khatak et al. 2020; Prasher et al. 2022). Additionally, inorganic carriers, particularly metal NPs like silver NPs, have demonstrated intrinsic antimicrobial characteristics (Sharma et al. 2021).

### 2.1 Drug Delivery in Pulmonary Infections

In general, for pulmonary infections, drugs are delivered to the lungs preferably by inhalation, where the drugs are nebulized into tiny particles before entering the lungs, as it takes advantage of the extensive surface area of alveoli, where rapid absorption takes place (Mishra and Singh 2020). However, conventional drug delivery is also used. For delivery of medicinal plants, there are a few dosage forms available, which are decoctions, tinctures, glycerites, herbal alcoholic beverages, capsules, and tablets. Similar to any chemically produced drug, the active constituent from the plant can be incorporated in most dosage forms as long as it is first extracted from the plant. Due to the multifaceted usage of plant metabolites, the dosage form required is based on the disease or the need of the patients (Kumadoh and Ofori‐Kwakye 2017). A plant and its constituents that are suitable for treating pulmonary infections exhibit several different pharmacological actions, including anti‐inflammatory and immunomodulatory action to antioxidative properties. These effects contribute to the regulation of inflammatory mediators, which hold potential in the treatment of pulmonary infections and supporting the body's natural defense mechanisms as well (Kamelnia et al. 2023). A diverse range of flexible nanomedicines is being developed, manufactured, and evaluated as carriers for inhaled drug delivery. Numerous bioactive compounds have been effectively enclosed within different nanomedicines for pulmonary drug delivery via inhalation (Gulati et al. 2021; Kumbhar et al. 2023).

### 2.2 COVID‐19

**FIGURE 3:**
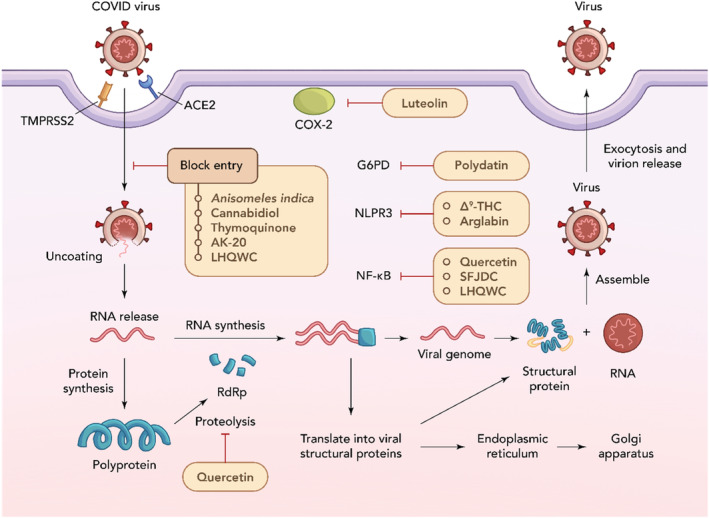
Mechanism of action of herbs that mitigate COVID‐19 infection.

SARS‐CoV‐2, or generally known as COVID‐19, had caused a worldwide pandemic in 2020. Two main entry pathways of SARS‐CoV‐2 were identified, which were angiotensin‐converting enzyme 2 (ACE2) through endosomal entry and through cell surface entry, which binds to transmembrane protease serine 2 (TMPRSS2) (Jackson et al. 2022). Research has looked at the possibility of probiotics, vitamins, amino acids, and flavonoids like curcumin as nutraceuticals to help reduce the symptoms of COVID‐19, such as fever, pain, and overall discomfort (Paudel, Patel, et al. 2022). When the SARS‐CoV‐2 virus fuses into the target cell, a cytokine storm will be triggered, including the release of various inflammasomes such as IL‐1, IL‐6, IL‐8, IL‐10, IFN‐γ, TNF‐α, monocyte chemoattractant protein‐1 (MCP‐1), CXCL‐10, and macrophage inflammatory protein‐1α (MIP‐1α) (Parasher 2021). Hence, to decrease the chances of COVID‐19 infection, a few plants have been identified to be capable of lowering the expression levels of ACE2 and TMPRSS2. Figure 3 schematically shows the various mechanisms of herbs that mitigate COVID‐19 infection. *Anisomeles indica* (L.) Kuntze, which has active constituents such as apigenin, ovatodiolide, and anisomlic acid, were found to have effects on lowering the expression of both ACE2 and TMPRSS2 in both in vitro (HepG2 and HEK 239T cell lines) and in vivo (mouse model) experiments. (Y.‐R. Chen et al. 2023).

*A*. *indica* is used as an ingredient in Jing Si herbal tea (JSHT), which is used as a complementary treatment used along with standard treatment in COVID‐19. In a clinical study, it was shown that this herbal tea can effectively decrease viral load of SARS‐CoV‐2, alleviate systemic inflammation, and enhance lung infiltrates in individuals with mild‐to‐moderate COVID‐19. No serious adverse effects were observed, with only four patients developing diarrhea after receiving treatment with JSHT. Hence, for cases of mild‐to‐moderate COVID‐19, JSHT as complementary treatment could be considered as a safe therapy (Hsieh et al. 2022).

Similarly, cannabidiol (CBD) extracted from *Cannabis sativa* was also found to lower ACE2 expression, hindering the replication of SARS‐CoV‐2. It decreased the viral load in lungs and reduced clinical disease signs in mice. However, further research would be required to understand the effects of cannabis smoke on lung injury that was caused by respiratory infections (Esposito et al. 2020; Preteroti et al. 2023; Zielińska et al. 2023). An in vitro experiment with human macrophages and primary human bronchial epithelial cells (HBECs) showed that not only CBD, but another compound found, Δ^9^‐tetrahydrocannabinol (THC), can inhibit NLRP3 inflammasome activation after cells were stimulated by LPS + ATP. CBD and THC were associated with decreased phosphorylation of STAT3, which correlated with reduced phosphorylation of tyrosine kinase‐2 (TYK2) (Suryavanshi et al. 2022). Furthermore, in inflamed mouse models, an experiment using various concentrations of extracts with high THC and CBD content was conducted. A specific high‐CBD extract, CBD‐X, which contains 35% CBD, 0.3% THC, and 0.3% CBG, was discovered to effectively raise anti‐inflammatory IL‐10 and reduce pro‐inflammatory cytokines. By reducing the migration of neutrophils to the lungs and decreasing the levels of IL‐1β, MCP‐1, IL‐6, and TNF‐α, the formation of cytokine storm is prevented (Aswad et al. 2022). Another study was done on producing a solid lipid nanoparticle (SLN) that is coated with tocilizumab (TCZ) and loaded with CBD. A synergistic effect is expected as TCZ functions as an anti‐IL‐6 receptor antibody for treating COVID‐19, which can reduce cytokine release syndrome. This SLN produced was observed to be able to protect the gastrointestinal tract from the detrimental effects of COVID‐19 (Zielińska et al. 2023). Dosage of CBD varies as various plasma levels of CBD may be necessary to trigger the different pathways responsible for its diverse effects. CBD has been evaluated in humans over a broad range, from < 1 to 50 mg/kg/day (Esposito et al. 2020). However, there are many side effects of CBD, which include aggravated respiratory symptoms such as coughing, sputum production, wheezing, and shortness of breath. Hence, further research would be required to understand the effects of cannabis smoke on lung damage resulted by respiratory infections (Preteroti et al. 2023). In short, CBD extracted has been shown to lower ACE2 expression, thereby hindering the replication of SARS‐CoV‐2. This reduction in viral load and clinical disease signs in mice highlights CBD's potential therapeutic effects against respiratory infections. However, further research is needed to explore the impacts of cannabis smoke on lung injury.

Vitamin D has a significant role in modulating the immune system's reaction in the body, as it can influence the production of antiviral peptides, thereby enhancing the body's natural defense mechanism (Ashique et al. 2023). Vitamin D2 can be found in mushrooms, while vitamin D3 are found in Solanaceae, Cucurbitaceae, Fabaceae, and Poaceae plant families, such as potatoes, bell peppers, and tomatoes (Hughes et al. 2018). Vitamin D works by upregulating ACE2 expression, which interestingly contradicts the medicinal plants above that downregulates ACE2 expression. This is because ACE2 expression regulates angiotensin II (AngII) expression, which induces lung injury and ARDS when accumulated. Hence, by increasing ACE2 and decreasing AngII expression, COVID‐19 can be less lethal, especially in elderlies with lower ACE2 expressions. Expression levels of ACE2 can be further increased when vitamin D is taken with IFN‐γ (Ashique et al. 2023; Coperchini et al. 2022). In another study, it was discovered that the active form of vitamin D, calcitriol, could impede the replication of SARS‐CoV‐2 in vitro. Mice that were fed with a diet rich with vitamin D (10,000 IU) showed improved resilience against acute respiratory damage and overall systemic issues, with reduced TNF, IL‐6, IL‐1β, and IFN‐γ levels along with an increased type I interferon (IFN) response (Campolina‐Silva et al. 2023). Hence, vitamin D plays a crucial role in modulating the immune system and enhancing the body's natural defense mechanisms through the production of antiviral peptides.

In a clinical study, patients with severe COVID‐19 who were treated with vitamin D (50,000 IU weekly) had a lower risk of death by Day 29. In human peripheral blood mononuclear cells (PBMCs), heightened activity was noted in the RIG‐1/MDA‐5 and JAK–STAT signaling pathways, in addition to noticeably higher levels of antiviral interferon stimulating genes (ISGs) including MX‐1 and ISG‐15 in both gene and protein expression. Similar observations were noted in both blood and saliva samples obtained from the same patients mentioned above (Hafezi et al. 2022).

*N*. *sativa* L. is a versatile herb and considered as a universal healer in Islam. Its bioactive constituents include α‐hederin, thymoquinone and thymohydroquinone. When *N*. *sativa* was first hypothesized to be a cure for COVID‐19, docking studies of its bioactive constituents showed that thymohydroquinone and α‐hederin had good binding energies toward ACE2 receptors. On the other hand, thymoquinone has anti‐inflammatory properties by inhibiting thromboxane B2 and leukotriene, which are products of oxidation from arachidonic acid. This inhibition occurs through the blocking of cyclooxygenase and lipoxygenase enzyme activities (Jakhmola Mani et al. 2020). Thymoquinone is able to decrease the levels of pro‐inflammatory agents such as IL‐2, IL‐4, IL‐6, and IL‐12, while boosting IFN‐γ. Not only that, but it also elevated the serum concentrations of lgG1 and lgG2a and improved the evaluation of pulmonary function in those with respiratory diseases that are restricted. Hence, it may be able to manage COVID‐19 by controlling overexpression of cytokines (Khazdair, Ghafari, and Sadeghi 2021). In vitro experiment using TQ has proven its potential to impede SARS‐CoV‐2 viral main protease with an IC50 value of 10.26 μM (Abdallah et al. 2022). In summary, *N*. *sativa* contains bioactive constituents that show potential against COVID‐19 by binding to ACE2 receptors and inhibiting inflammatory pathways. Thymoquinone, in particular, reduces pro‐inflammatory agents and enhances immune response, helping manage cytokine overexpression and inhibiting SARS‐CoV‐2 main protease.

Clinical trials using *N*. *sativa* to treat COVID‐19 had shown some effects. A study using 500 mg of *N*. *sativa* oil (NSO) twice daily on patients with mild COVID‐19 infection had shown that the number of recovered patients (62%) from using NSO was higher than control group (36%), with a shorter recovery duration (Koshak et al. 2021). Another study using *N*. *sativa* with honey (HNS) had successfully decreased the duration needed to alleviate COVID‐19 symptoms in both moderate and severe cases by half (Ashraf et al. 2023). However, the dosage of *N*. *sativa* did not make a large difference in symptoms, differentiation profile and inflammatory marker in COVID‐19 patients (Bin Abdulrahman et al. 2022).

Similar to thymoquinone, arglabin extracted from *Artemisia glabella* also works against COVID‐19 by preventing the formation of cytokine storms. It inhibits NLRP3 inflammasome, suppresses synthesis of IL‐2, IL‐1β, IL‐18, and TNF‐α, which contributes to morbidity in COVID‐19. Arglabin is suggested for use in more critical cases of COVID‐19. However, additional research is required to prove the safety and toxicology profile of arglabin (Manayi et al. 2021). *Pimenta dioica* (L.) Merr with its four main bioactive constituents, which are rutin, gallic acid, chlorogenic acid, and ferulic acid, were found to be able to reduce the levels of TNF‐α, G‐CSF, IL‐1β, IL‐2, and mRNA155 gene expression in HgCl_2_‐treated mice. Not only that, it was also associated with increase in IL‐10 levels and expression of mRNA21‐3p gene. Among the bioactive constituents, chlorogenic acid, rutin, and gallic acid exhibited potent anti‐SARS‐CoV‐2 activities with high SARS‐CoV‐2 inhibitory concentrations in vitro, while ferulic acid (50 mg/kg) and rutin (75 mg/kg) had better anti‐inflammatory effects in vivo. Rutin was suggested as the compound to have further research done on managing COVID‐19 (El Gizawy et al. 2021). In short, arglabin from *A*. *glabella* and the bioactive constituents of *P*. *dioica* show potential against COVID‐19 by preventing cytokine storms.

Quercetin or its derivatives, which can be found in the extract of *Ginkgo biloba* L. (EGb), can inhibit 3‐chymotrypsin‐like protease (3CLpro) and papain‐like protease (PLpro). 3CLpro and PLpro are required in SARS‐CoV‐2 replication, where it cleaves the newly synthesized polypeptide chain, producing various nonstructural proteins (NSPs) necessary for viral replication (Mody et al. 2021). Additionally, quercetin can successfully reduce inflammation and oxidative stress by blocking the NF‐κB signaling pathway. Not only that, SARS‐CoV envelope protein E can be efficiently inhibited by kaempferol and quercetin with their potent channel‐blocking activity. EGb has the capacity to impede the progression of inflammatory lung conditions, potentially attenuating acute lung injury and respiratory complications in COVID‐19. EGb extract NPs with a dosage of 40 mg/kg have been developed to improve its oral bioavailability (L. Wang et al. 2019). It has been suggested that EGb, when combined with vitamin C, can be used as an effective combination to avert and address COVID‐19 (Al‐Kuraishy et al. 2022). Acute toxicity studies have demonstrated that EGb has a lethal dose of 7.73 g/kg in mice when administered orally. EGb has shown bleeding as its adverse effects in various case studies, but there were also cases where there was no bleeding as an adverse effect. Hence, there is insufficient evidence and further research to understand the adverse effects of EGb is required (Akanchise and Angelova 2023). In conclusion, quercetin and its derivatives from *G*. *biloba* extract inhibit key proteases essential for SARS‐CoV‐2 replication and reduce inflammation by blocking the NF‐κB pathway.

*Achillea millefolium* with luteolin as its bioactive constituent can inhibit or down‐regulate cyclooxygenase II (PTGS2), which is anti‐inflammatory. It also has antiviral activity, where it can disintegrate the virus membrane of SARS‐CoV‐2 cells. However, to determine the mechanism behind this constituent's antiviral and anti‐inflammatory activities, additional research is needed (Tilwani et al. 2023). In patients in severe stages of COVID‐19, a different strategy can be used as a complementary approach to enhance the condition of individuals by targeting glucose‐6‐phosphate dehydrogenase (G6PD) using polydatin, a glycoside form of resveratrol. It can inhibit G6PD to limit oxidative damage and improves airway inflammation, preventing the formation of cytokine storm. Polydatin can be found not only in *Polygonum* but also in many food items including peanuts, grapes, red wine, and cocoa (Doustimotlagh and Eftekhari 2021). For patients experiencing post‐COVID‐19 symptoms, ginsenosides from *Panax notoginseng* offers new approaches as it can impact virus‐related tissue damage, inflammation, immune response, and other factors, helping relieve respiratory and pulmonary symptoms, reducing cardiac stress, and safeguarding the nervous system. It can block the triggering of signaling pathways involving MAPKs, NF‐κB, and c‐Fos (Y. Wang et al. 2023). Hence, it can be seen that there are medicinal plants for long‐term and post‐COVID‐19 symptoms, but further research would be required to ensure their efficacy and adverse effects.

Other than individual plants, there are also folk medicine used, produced with a combination of plants to combat COVID‐19. In Ayurveda, a decoction called Arogya Kashayam (AK‐20) is used. It is a powder made from the stem of *Tinospora cordifolia*, rhizome of *Zingiber officinale* Roscoe, whole plant of *Phyllanthus niruri* Linn., root of *Glycyrrhiza glabra* Linn., fruits of *Terminalia chebula* Retz., *Piper longum* Linn., and *Piper nigrum* Linn. In this study, patients treated with AK‐20 alone had a 93% rate of recovery, while patients treated with a combination of AK‐20 and hydroxychloroquine (HCQ) had a 94.17% rate of recovery (Shukla et al. 2019). A key component of AK‐20 was *Glycyrrhiza glabra*, or known as liquorice, with its main bioactive constituent of glycyrrhizin, is found to be able to bind to ACE2, inhibiting entry of the SARS‐CoV‐2 virus. A study showed that ACE2 expression had significantly decreased in healthy individuals after 7 days of 50 g of liquorice intake. Not only that, HMGB1 levels, which is involved in inflammation, were decreased as well (Buder et al. 2022). Statistically, the current investigation demonstrates that the combination of AK‐20 with HCQ yielded superior outcomes, with 85% of cases exhibiting negative RT‐PCR conversion within 10 days compared to HCQ alone where 75% achieved negative RT‐PCR results in the same time frame, with no serious adverse effects. Notably, recent research highlights the role of elevated ferritin levels (hyperferritinemia) in exacerbating COVID‐19 severity, acting as a crucial mediator of immune dysregulation and pro‐inflammatory effects. Both groups in this study exhibited a notable decrease in serum ferritin levels. It is crucial to highlight that elevated cellular iron and hemoglobin levels correlate with an elevated risk of severity in COVID‐19 cases (Shukla and Ujjaliya 2023). Last but not least, AK‐20 is highly effective against COVID‐19, particularly when combined with HCQ, achieving a 94.17% recovery rate.

Another formulation, Shufeng Jiedu capsule (SFJDC) from China, was also used in treatment of COVID‐19. It consists of *Verbena officinalis*, *Fallopia japonica*, *Forsythia suspensa*, *Isatis indigotica*, *Glycyrrhiza uralensis*, *Patrinia scabiosaefolia*, *Phragmites australis*, and *Bupleurum chinense*. These plants exert a synergistic effect with each other. The bioactive constituents include saikosaponins, resveratrol, emodin, forsythoside A, glycyrrhizin, indirubin, vitexin and quercetin. SFJDC has the capability to mitigate stress‐induced injury triggered by LPS and hinder inflammation in lung tissue by inhibiting the signaling pathway of MAPK and NF‐κB. (Y. Xu et al. 2022) In the HCoV‐229E mouse model, SFJDC was examined, revealing a substantial decrease in lung viral load accompanied by lowered IL‐6, IL‐10, TNF‐α, and IFN‐γ levels (Xia et al. 2021). Hence, SFJDC shows potential as a therapeutic option for COVID‐19, but its adverse reactions would require further research and larger clinical studies with double‐blind, randomized controlled trials would be needed (Xia et al. 2021; Y. Xu et al. 2022). In summary, SFJDC shows promise for COVID‐19 treatment, with studies indicating its ability to reduce lung injury, inflammation, and viral load. However, further research, including larger clinical trials, is needed to confirm its effectiveness and safety.

Furthermore, another formulation from China, known as Lianhuaqingwen (LHQW), was explored for its usage in treating COVID‐19. It was developed and approved as a treatment for various public crises, including SARS, H3N2, and H1N1 influenza, and is usually delivered through tablet, capsules, or granules form (Liang et al. 2021). Its formulation includes 13 different herbs, which are *Forsythia suspensa*, *Lonicera japonica*, *Isatis indigotica*, *Dryopteris crassirhizoma*, *Houttuynia cordata*, *Ephedra sinica*, *Armeniaca sibirica*, *Pogostemon cablin*, *Rhodiola rosea*, *Glycyrrhiza uralensis*, *Rheum officinale*, and *Mentha haplocalyx*. The main compounds found that could target ACE2 receptor were forsythoside A, forsythoside I, rhein, glycyrrhizin, amygdalin, neochlorogenic acid, rutin, and prunasin (X. Chen et al. 2021). Not only those, but compounds such as quercetin, forsythoside E, hyperoside, rutin, and kaempferol could also be found in LHQW, where they have high binding energies toward 3CLpro (Liang et al. 2021; Ling 2020). An in vitro experiment done by Beijing Yiling Pharmaceutical Co. Ltd. using MDCK cells showed that LHQW had a half‐toxic dose of 1.02 mg/mL, while an in vivo experiment using chicken embryo showed that no harmful effects could be seen with 1 g/mL of drug dosage used.

A meta‐analysis of clinical studies conducted for LHQW showed its effectiveness in treating COVID‐19, with no serious adverse effects reported (K. Hu et al. 2021; M. Liu et al. 2021). However, it was observed that LHQW seems to have higher efficacy when taken in conjunction with standard treatment (M. Liu et al. 2021). Hence, it can be seen that LHQW does have some effect on treating COVID‐19, as the compounds in this formulation can prevent entering and replication of COVID‐19 virus. Table 1 shows the summary of all plants mentioned in this segment.

**TABLE 1:** A summary of plant species and mixtures and their bioactive constituents that have effect on treating and preventing COVID‐19 infection.

Plant	Active constituent	Method of delivery	Properties	References
*Anisomeles indica* (L.) Kuntze	Apigenin, ovatodiolide, anisomlic acid	Oral	Reduce expression levels of ACE2 and TMPRSS2 in vitro and in vivo with a dosage of 500 mg/kg. Used in Jing Si herbal tea, effectively decreased viral load of SARS‐CoV‐2.	Y.‐R. Chen et al. 2023; Hsieh et al. 2022
*Cannabis sativa*	Cannabidiol (CBD) Δ^9^‐tetrahydrocannabinol (THC)	Intravenous or inhalation	CBD lowers ACE2 expression, hindering replication of SARS‐CoV‐2. THC inhibits NLRP3 inflammasome activation. Both decrease phosphorylation of STAT3.	Esposito et al. 2020; Preteroti et al. 2023; Suryavanshi et al. 2022; Zielińska et al. 2023
Solanaceae, Cucurbitaceae, Fabaceae, and Poaceae families	Vitamin D, calcitriol	Oral	Upregulates ACE2 expression, decreases lethality of COVID‐19 at a dosage of 50,000 IU weekly. Increases RIG‐1/MDA‐5 and JAK–STAT signaling pathways. Calcitriol can impede replication of SARS‐CoV‐2 in vitro.	Ashique et al. 2023; Campolina‐Silva et al. 2023; Coperchini et al. 2022; Hafezi et al. 2022
*Nigella sativa* L.	Thymoquinone, thymohydroquinone, α‐hederin	Oral	Thymohydroquinone and α‐hederin have good binding energies toward ACE2 receptors. Thymoquinone can decrease levels of pro‐inflammatory agents and manage COVID‐19 by controlling overexpression of cytokines.	Abdallah et al. 2022; Jakhmola Mani et al. 2020; Khazdair, Ghafari, and Sadeghi 2021
*Artemisia glabella*	Arglabin	—	Prevents formation of cytokine storms and inhibits NLRP3 inflammasome.	Manayi et al. 2021)
*Pimenta dioica* (L.) Merr	Rutin, gallic acid, chlorogenic acid, ferulic acid	Oral	Rutin, gallic acid, and chlorogenic acid exhibited potent anti‐SARS‐CoV‐2 activities with high SARS‐CoV‐2 inhibitory concentrations in vitro, while ferulic acid (50 mg/kg) and rutin (75 mg/kg) had better anti‐inflammatory effects in vivo.	El Gizawy et al. 2021
*Ginkgo biloba* L.	Quercetin	Intravenous	Inhibit 3‐chymotrypsin‐like protease and papain‐like protease, blocks NF‐κB signaling pathway. Can impede progression of inflammatory lung conditions.	Al‐Kuraishy et al. 2022
*Achillea millefolium*	Luteolin	—	Inhibit or downregulate cyclooxygenase II.	Tilwani et al. 2023
*Polygonum*	Polydatin	Oral	Targets glucose‐6‐phosphate dehydrogenase (G6PD), limits oxidative damage, and improves airway inflammation.	Doustimotlagh and Eftekhari 2021
*Panax notoginseng*	Ginsenosides	Oral	For post‐COVID‐19 symptoms, relieve respiratory and pulmonary symptoms, block triggering of signaling pathways.	Y. Wang et al. 2023
Arogya Kashayam (AK‐20)	Glycyrrhizin	Oral	Lowers ACE2 expression and HMGB1 levels. AK‐20 with HCQ can treat COVID‐19 within a shorter time frame.	Shukla et al. 2019; Shukla and Ujjaliya 2023
Shufeng Jiedu capsule (SFJDC)	Saikosaponins, resveratrol, emodin, forsythoside A, glycyrrhizin, indirubin, vitexin, quercetin	Oral	Mitigate stress‐induced injury triggered by LPS and hinder inflammation in lung tissue by inhibiting signaling pathway of MAPK and NF‐κB. Lowers viral load in vivo.	Xia et al. 2021; Y. Xu et al. 2022
Lianhuaqingwen (LHQW)	Forsythoside A, forsythoside I, rhein, glycyrrhizin, amygdalin, neochlorogenic acid, rutin, prunasin, quercetin, forsythoside E, hyperoside, and kaempferol	Oral	Targets ACE2 receptor and binds to 3CLpro, preventing entry and replication of virus.	X. Chen et al. 2021; Liang et al. 2021; M. Liu et al. 2021

### 2.3 Influenza

Influenza A or B virus is the source of influenza, an acute respiratory illness (Majumder et al. 2022; Moghadami 2017; Shastri et al. 2021). Three different virus types can cause influenza which are A, B, and C. Although Type A viruses have many antigen‐based subgroups, Type B and Type C viruses do not (V. Kumar 2017). Infections caused by IAV trigger heightened antiviral reactions and induced inflammatory responses within primary bronchial epithelial cells (pBECs) derived from both nonsmoking individuals and smokers (Hsu et al. 2017). Three different pathways can be activated by viral RNA, which are Nod‐like receptors (NLR), Toll‐like receptors (TLR), and retinalin‐induced gene‐1 (RIG‐1) protein. IL‐1β and IL‐18 are released, which would be matured after activation of NLPR3 (Devkota et al. 2021; Patel et al. 2022). IL‐18 triggers IFN‐γ production, while IL‐6 is induced with IL‐1β. Furthermore, overproduction of TNF‐α can cause a cytokine storm as NF‐κB, MAPK, and STAT3 signaling pathways are excessively activated (Gu et al. 2021). Influenza frequently happens in global outbreaks and epidemics, particularly in the winter. Sneezing and coughing can spread an infection through big particle droplets (Moghadami 2017). The 2009 pandemic brought on by a new H1N1 virus with swine origins serves as evidence that this disease is significant for global public health. Annually, influenza outbreaks cause significant illness and school absenteeism among healthy children and adolescents. Its genetic makeup can be altered, making it susceptible to diseases with pandemic potential (Labella and Merel 2013). The early signs and symptoms of influenza usually include a high‐grade fever, myalgia, headache, and malaise. Respiratory tract symptoms such as sore throats, nonproductive coughs, and nasal discharge accompany these presentations (Moghadami 2017). However, influenza virus infection can cause acute respiratory failure and pneumonia, which are often made worse by coinfection with bacteria (Peteranderl, Herold, and Schmoldt 2016). The maximum rates of hospitalization and infection in influenza among pediatrics are generally seen in infants and young children. Potential contributing factors include the immunodeficiency of the infant, previous immunity deficient as well as viral exposure (Munoz 2003).

Vaccines and antiviral medications are examples of preventative measures (Talbot 2017). Chemical and pharmacological research has led to the isolation of several anti‐influenza medicines from plants, but the mainstays of influenza control and therapy remain chemical or biochemical therapies. Numerous flavonoids, polyphenols, saponins, alkaloids, and glucosides are among these agents. Traditional medicinal drug emphasizes on medicinal plants' therapeutic use, and TCM has achieved improvement in both clinical trials and management of influenza with associated symptoms (X. Wang et al. 2006).

For instance, *Rapanea melanophloeos* (RM) is categorized under family *Myrsinaceae*. The medicinal part of this plant used is leaf. Studies showed that *R*. *melanophloeos* has remarkable antiviral potential against the Influenza A virus. In South Africa, it has been used for treatment of TB‐related symptoms. A bioactive component which is useful in the plant to treat influenza is quercetin‐3‐O‐α‐L‐rhamnopyranoside (Q3R). Alkaloids, flavonoids, tannins, saponins, terpenoids, cardiac glycosides, and phlobatannins were all present in RM. When it came to TNF‐α concentration, the inoculation of virus produced a significant level of pro‐inflammatory cytokines, but this protein showed decreases in all combination treatments with RM, particularly in the co‐penetration treatment. In terms of IL‐27 concentration, all combination treatments considerably raised the level of IL‐27 protein in comparison to virus inoculation (Mehrbod et al. 2018). Research has shown that IL‐27 will induce anti‐inflammatory activity via the stimulation of the production of CD4, CD8 T cells, and NKT cells (Carl and Bai 2008). Besides, the most common route of administration was oral (Amenya et al. 2014). The chloroformic extract from the stem bark of RM led to a notable decrease in bodyweight when administered at 1000 mg/kg in animal trials. Nonetheless, the blood analysis revealed raised levels of red blood cells and hematocrit, coupled with reduced mean corpuscular hemoglobin and mean corpuscular hemoglobin concentration. Despite these findings, the extract exhibited no signs of toxicity at doses up to 1000 mg/kg, indicating its potential suitability for use in African traditional medicine at similar or lower concentrations (Amenya et al. 2014). In short, Q3R from RM shows promise in mitigating pro‐inflammatory cytokine levels while inducing anti‐inflammatory responses, suggesting its potential as a therapeutic agent.

*Canarium album* is classified under the Burseraceae family. *Ganlanye* (GLY), the leaves of the *C*. *album* is mainly used to treat influenza. From the GLY aqueous extract, the extraction of three novel phenolic glycosides, canaroleosides A–C (1–3), along with three familiar flavonoids (4–6), led to the discovery of their mechanism for combating influenza viruses (Xiao et al. 2022). GLY is mainly utilized to treat Influenza A. It was discovered that six bioactive ingredients were found to have anti‐influenza virus action, among which are canaroleosides A–C (1–3). Because of the two gallates' distinct locations and the 1,2,4‐trisubstitutedphenyl moiety, canaroleosides A and C able to inhibit neuraminidase activities with higher effectiveness than canaroleoside B. This suggests the significance of the substituents at glucosyl's positions C‐1 and C‐2. Canaroleosides A, B, and C were predicted by molecular docking to be able to enter NA's groove and establish a hydrogen bond to bond with the amine side chain of arginine. Moreover, canaroleoside A containing substituents at the positions of glucosyl's C‐1, C‐2, and C‐3 may prevent hemagglutination (Rakers et al. 2014; Sadati et al. 2019). Under the category of Burseraceae family, no deaths were observed during a 14‐day period of acute oral toxicity testing. There were also no notable gross or histopathological abnormalities found in the liver or kidneys. The LD50 (lethal dose for 50% of the test subjects) of the ethanolic extract in mice was determined to be greater than 5 g/kg body weight (Arenas and Trinidad 2017). In summary, *C*. *album*, specifically its leaves GLY, exhibits promising potential in treating Influenza A, attributed to bioactive compounds like canaroleosides A–C.

In a similar study, tiliroside extracted from Fu Rong Ye (FRY), which is the leaves of *Hibiscus mutabilis L*. is studied on its effects against influenza. Tiliroside is a glycosidic flavanoid, where it suppressed viral growth of various strains of IAV in a multicycle growth inhibitory assay. It also targets viral ribonucleoprotein (RNP), which is essential with viral genome replication as well as transcription. Furthermore, it reduces the expression of cytokines and chemokines in A549 cells. Infected mice were treated with 800 mg/kg/d of tiliroside, where 50% of mice lived and their lung edema conditions were improved (Xiao et al. 2023). Another study was conducted on total alkaloids extract (TA) obtained from *Alstonia scholaris*, where the four main bioactive constituents are scholaricine, picrinine, 19‐epischolaricine, and vallesamine. The anti‐inflammatory properties of the plant were attained by the activation of β2 adrenergic receptor and inhibition of NF‐κB expression. An in vivo study involving mice infected with H1N1 revealed that a minimum dose of 12.5 mg/kg TA could lengthen the survival period of the infected mice, while an in vitro experiment with chicken embryos demonstrated that the presence of TA suppressed H1N1 virus replication (Zhao et al. 2021). However, there are insufficient clinical studies for toxicology and adverse reactions.

Pomegranate which is extracted from *Punica granatum* serves as a viral replication suppressant and potent inhibitor, impeding the multiplication of viruses within host cells. Its virucidal properties enhance effectiveness by directly targeting and inactivating influenza viruses (Mousa 2017). The primary anti‐inflammatory components in pomegranate extract, namely ellagic acid and ellagitannins, play a crucial role. Ellagitannins not only prevent the synthesis of various inflammatory indicators but also reduce their promoter inhibition. Upon breakdown by gut microbiota, they produce urolithins, with urolithins being the primary ingredient believed to be responsible for pomegranate's anti‐inflammatory qualities. This is attributed to the proposed reduction in PGE2 synthesis, hindering NF‐κB activation, downregulating MAPK, and suppressing COX‐2 and mPGES‐1 expression (González‐Sarrías et al. 2010). Hence, the anti‐inflammatory effect can be achieved via the prevention of pro‐inflammatory cytokines production, such as IL‐1β as well as TNF‐α (H. Yu et al. 2020). The involvement of neutrophils in inflammatory processes is emphasized due to their substantial reactive oxygen species (ROS) release produced by NADPH oxidase and myeloperoxidase (Zarfeshany, Asgary, and Javanmard 2014). The comminuted herbal substance is added to water in the form of a decoction, infusion, or maceration, and the powdered drug is administered orally (Dioguardi et al. 2022). Despite the absence of safety concerns associated with the doses administered in clinical studies, it is important to acknowledge that pomegranate preparations might have adverse effects by stimulating the metabolism of synthetic drugs through the activation of liver enzymes (Vlachojannis, Zimmermann, and Chrubasik‐Hausmann 2015). In conclusion, pomegranate extract from *P*. *granatum* exhibits potent antiviral properties, particularly against influenza viruses, by suppressing viral replication and directly inactivating viruses.

*Epimedium koreanum* Nakai has remarkable potential in reduction in viral replication, heightened type I IFN secretion, pro‐inflammatory cytokines, and immunomodulation (Mousa 2017). Abundant bioactive constituents found in *E*. *koreanum* are flavonoids such as quercetin, icariin, epimedosides, hyperoside, icariside II, epimedin and chlorogenic acid (Yasmin et al. 2020). Flavonoids show antimicrobial, anti‐inflammatory, and antioxidant activity (Qian et al. 2024). The antiviral activity of *E*. *koreanum* was only recently discovered (Cho et al. 2012). Aqueous extracts of *E*. *koreanum* exhibits heme oxygenase‐1 (HO‐1) expression, reducing iNOS and COX‐2 expression, suppressing NO, IL‐1β, and IL‐6 production substantially, and these compounds prevent the phosphorylation and degradation of κBα, inhibit the nuclear translocation of NF‐κB, and hinder its binding to DNA in LPS‐stimulated RAW 264.7 cells. Additionally, ikarisoside A has a synergic effect with the combination of *E*. *koreanum* (Qian et al. 2024). Through both in vitro and in vivo animal models, Cho et al. demonstrated a potent antiviral effect of the aqueous extract of *E*. *koreanum* to combat the PED virus. Test results indicated that *E*. *koreanum* modulates immunological responses, including lymphocyte and macrophage activation, thereby exhibiting an antiviral impact (Cho et al. 2012). For adverse reactions, the hepatotoxicity of *E*. *koreanum* Nakai ethanol extract (EEE) is not well understood. Experiments on rats revealed that exposure to EEE for 28 days resulted in increased liver weight and elevated levels of liver enzymes in serum, indicating significant liver damage. Additionally, severe cytoplasmic vacuolation was observed in liver tissue. Metabolomics analysis showed notable changes in liver and serum metabolites, suggesting EEE‐induced liver injury. Certain metabolites in serum, such as flavin mononucleotide, phenylacetylglycine, glutathione, L‐tryptophan, and sphingomyelin, were identified as potential markers for EEE‐induced liver injury (P. Li et al. 2022). In summary, *E*. *koreanum* Nakai demonstrates potent antiviral activity, reducing viral replication and enhancing type I IFN secretion while modulating pro‐inflammatory cytokines. However, caution is warranted due to the potential hepatotoxicity observed in animal studies, suggesting the need for further investigation into its safety profile. Another study concluded that *E*. *koreanum* Nakai is effective as an immunomodulator and shows promise as a prophylactic or therapeutic treatment against a range of viruses in animals and humans.

*S*. *baicalensis* Georgi, specifically its active compound baicalin, functions as a neuraminidase inhibitor, hindering the enzymatic activity of neuraminidase and thereby preventing virus budding. Classified as a flavonoid, baicalin was isolated from *Radix scutellaria* and may be able to suppress the H1N1 and H3N2 viruses in A549 cells (Mousa 2017). It was revealed that baicalin therapy significantly minimizes miR‐146a expression. Hence, it results in the suppression of specific pro‐inflammatory chemokines, such as C‐X‐C motif chemokine ligand (CXCL)1 and IL‐8, and decreased activity of NF‐κB pathway (Kivihall et al. 2019). miR‐146a expression involves targeting TNF receptor‐associated factor 6 (TRAF6), an essential adapter in the IFN production signaling pathway. The downregulation in the production of type I IFN triggered by miR‐146a contributed to enhanced viral replication. Moreover, enrichment of miR‐146a was discovered to eliminate the anti‐influenza virus A (IVA) effects of baicalin on both H1N1 and H3N2 viruses (R. Li and Wang 2019). Furthermore, in A549 cells infected with H1N1 or H3N2, baicalin significantly reduced miR‐146a expression. The introduction of a miR‐146a mimic disrupted baicalin's antiviral activities, suggesting that baicalin influences miR‐146a. Subsequent tests identified TRAF6 as a direct target of miR‐146a during IVA infection. The miR‐146a mimic inhibited the type I IFN response triggered by TRAF6. In vivo tests demonstrated baicalin's protective effect against H1N1 infection through miR‐146a inhibition (Yoshida et al. 2008). In vivo studies further supported the potential of baicalin to shield mice from H1N1 infection, emphasizing its therapeutic promise in combating influenza infections. In a study, *S*. *baicalensis* Georgi exhibited toxicity primarily in the form of liver fibrosis and allergic reactions, largely attributed to baicalin. However, there is currently insufficient clinical research available on this matter (Song et al. 2020). In short, baicalin therapy suppresses pro‐inflammatory chemokines such as CXCL1 and IL‐8, along with the NF‐κB pathway, by downregulating miR‐146a expression. This downregulation enhances the production of type I IFN and contributes to baicalin's antiviral effects. However, concerns regarding liver fibrosis and allergic reactions associated with baicalin highlight the need for further clinical research to fully understand its safety profile.

*Paeonia lactiflora* Pallas (Bai Shao) acts as an inhibitor of viral RNA and viral protein synthesis, while also disrupting viral hemagglutination. Its mechanism involves interference with viral binding to and penetration into host cells (Mousa 2017). Additionally, the bioactive compound paeoniflorin (Pae), a monoterpene glucoside isolated from *P*. *lactiflora*'s roots, has demonstrated robust anti‐inflammatory and anti‐fibrotic properties (X. Yu et al. 2019). Pae lowers the synthesis of inflammatory media, modifies immune cell activation and function, and repairs aberrant signal pathways. By suppressing aberrantly cell subsets activation and reestablishing regulatory cell subsets, Pae may be able to restore immunological cell subset balance. Pae may regulate the GPCR pathway; the TGFβ with Smads pathway; the MAPKs with NF‐κB pathway; the PI3K, Akt, and mTOR pathway; the JAK2 with STAT3 pathway; and other signaling pathways (L. Zhang and Wei 2020).

Yang et al. (2012) conducted a randomized controlled trial over 3 months comparing the efficacy of total glucosides of peony capsule (TGP) and compound glycyrrhizin tablet for alopecia areata. They enrolled 86 outpatients who were randomly assigned to two groups. The TGP group, consisting of 44 patients, received 10 mg vitamin B2 and three doses of 600 mg TGP daily. The control group received three doses daily of 50 mg glycyrrhizin. In the TGP group, 12 adverse reactions were reported, including two cases of abdominal pain, six cases of loose stool, and four cases of increased stool frequency. In contrast, the glycyrrhizin group experienced 14 adverse reactions, such as two cases of hypokalaemia, three cases of increased blood pressure, five cases of edema, two cases of increased weight, and one case of decreased muscle strength (D.‐Q. Yang et al. 2012). In summary, *P*. *lactiflora* Pallas (Bai Shao) demonstrates antiviral and anti‐inflammatory effects, while its active compound, paeoniflorin, has shown efficacy in conditions like alopecia areata with manageable adverse reactions.

Essential oils (EOs) are diverse compounds derived from plants, possessing therapeutic properties effective against infectious diseases, including influenza. They can function as inhibitors at various stages of influenza virus prevalence including genome replication, binding, penetrating, uncoating, assembly, and virus release (Oriola and Oyedeji 2022). An example would be *Melaleuca alternifolia* or commonly known as tea tree oil. Its bioactive constituents such as terpinen‐4‐ol and α‐terpineol can inhibit the entry and fusion of the influenza virus and disrupt the acidification process of intralysosomal compartment. (Madia et al. 2022) Another notable plant would be *Curcuma longa*, where the bisabolane‐type sesquiterpenoids that are extracted can inhibit replication in MDCK and A549 cells infected with H1N1. Not only that, the compounds suppresses the production of pro‐inflammatory mediators such as TNF‐α, IL‐6, IP10, and IL‐8, while modifying the activity of RIG‐1/STAT‐1/2, and NF‐κB/MAPK signaling pathways (Ti et al. 2021). Eugenol extracted from *Cinnamomum zeylanicum* and citronellol from *Pelargonium graveolens* can act against influenza as both compounds act as neuraminidase inhibitor, concentrating on the viral surface before and during the stage of adsorption in the virus's lifetime (Oriola and Oyedeji 2022). However, there are insufficient studies on toxic dose and safety profile.

In zedoary oil produced from *Curcuma zedoaria* that was used in TCM, three main constituents are found, including curcumol, curdione, and germacrone, by which germacrone was found to be the compound with the strongest anti‐influenza effect. It showed the ability to stimulate the IFN genes transcription, shielding the peripheral cells away from influenza virus infections. It exhibited a significant reduction in antiviral proteins expressions such as RIG‐I, IFNs, OAS, IRF3/7, MX, and EIF2AK2/PKR. Additionally, there was a decrease in viral replication and its load, accompanied with heightened TAP1 expression. Germacrone blocked TAK1 phosphorylation leading to suppression of NF‐κB signaling and intrinsic antiviral responses. (L. Li et al. 2020) Furthermore, a popular EO would be eucalyptus oil, which contains eucalyptol. It was found to lower cytokine levels in fluid samples collected from nasal passages and levels of IL‐1β, IL‐6, TNF‐α, and IFN‐γ in influenza A‐infected mice. Not only that, eucalyptol exhibits protective effects against influenza virus by mitigating inflammatory reactions within pulmonary tissues (Ait‐Ouazzou et al. 2011). Camphecene, which is a camphor derivative, was found to be a HA inhibitor, which can lower viral pathogenicity (Zarubaev et al. 2018). According to the findings, the LD50 value for intraperitoneal injection of *C*. *zedoaria* EO was determined to be 1.76 mL/kg of body weight, indicating the dose at which 50% of the test subjects experienced lethality. Additionally, the maximum nonfatal dose was identified as 0.96 mL/kg of body weight, suggesting the highest dose administered that did not result in fatality (Mahmoudvand et al. 2020). In summary, zedoary oil from *C*. *zedoaria* contains curcumol, curdione, and germacrone, with germacrone exhibiting potent anti‐influenza effects by stimulating IFN gene transcription, inhibiting viral replication, and reducing viral load.

SFJDC was also found to be able to treat influenza virus. When SFJDC (0.6 g/kg/day) was combined with oseltamivir (25 mg/kg/day) in vivo, it was found to be able to downregulate IL‐1β and IL‐18 levels in serum and BALF of rats and decreased the expression of NLPR3‐associated components. This combination had shown significant synergistic benefits with few to no adverse effects and reduced drug resistance compared to oseltamivir alone, likely due to SFJDC's inhibitory effects on viral proliferation, immunoregulatory functions, and anti‐inflammatory properties. However, further clinical studies and studies in a larger scale would be needed to evaluate the effectiveness and mechanism of this combination (Ji et al. 2020). As for LHQW capsule, studies have found that it can inhibit influenza replication and inflammatory cytokines in both in vitro and in vivo experiments. In virally infected mice, administration of 650 and 1300 mg of LHQW solution reduced inflammatory cytokines such as TNF‐α and IL‐6. Furthermore, neither dosage produced adverse effects in the mice (Ding et al. 2017; X. Shen and Yin 2021).

In a systematic review and meta‐analysis of SFJDC, it was found that combining SFJDC with conventional western medicine decreased the time needed for symptoms of influenza to be relieved and shortened the time for cough to improve. Moreover, there were also no significant differences in adverse effects in this combination as well. However, better quality studies can be included with larger sample sizes (Zhou et al. 2023). On the other hand, meta‐analysis of LHQW indicated that it was more effective in relieving flu symptoms compared to oseltamivir, while it had a higher effect on temperature reductions compared to ribavirin and Ankahuangmin capsules (Niu et al. 2017). Another meta‐analysis had analyzed the adverse effects of LHQW capsules when treating influenza A, where there was a significantly lower incidence of adverse reactions with LHQW compared to conventional drugs. However, further studies that are long‐term and high‐quality are needed (C. Hu et al. 2022).

Comparative studies between herbs and standard treatments for influenza has been evaluated. Quercetin, which can be found in *R*. *melanophloeos* and *E*. *koreanum* Nakai, was tested in docking studies, where it had demonstrated a strong potential and high affinity for binding to the active site of NA domain N1, exhibiting lower binding energies (−6.8 kcal/mol for quercetin and − 5.8 kcal/mol for oseltamivir) (Mehrbod et al. 2021). Another study comparing quercetin 3‐glucoside (Q3G) and oseltamivir in vitro demonstrated that Q3G had higher anti‐influenza activity in all influenza strains tested, whereas oseltamivir had weaker activity on some A and B influenza strains. Furthermore, Q3G showed higher virus blocking activities compared to oseltamivir, indicating its effective inhibition of the influenza virus (Nile et al. 2020). In a systematic review of medicinal plants, no significant difference was found between the use of medicinal plants and oseltamivir individually. However, when used together, there was a significant reduction in fever duration by 7.84 h, without an increase in adverse effects. This indicates that combining medicinal plants with oseltamivir can enhance the therapeutic effect (Choi, Lee, and Chang 2020). Table 2 shows a summary of all the plants mentioned in this segment.

**TABLE 2:** A summary of plant species and their constituents that have effect on treating and preventing influenza A infection.

Plant	Active constituent	Method of delivery	Properties	References
*Rapanea melanophloeos*	Quercetin‐3‐O‐α‐L‐rhamnopyranoside	Oral	Increase IL‐27 levels, inducing anti‐inflammatory activity.	Carl and Bai 2008; Mehrbod et al. 2018
*Canarium album* (Ganlanye)	Canaroleosides A‐C, flavanoids	—	Inhibit neuraminidase activity, has anti‐influenza virus action.	Rakers et al. 2014; Sadati et al. 2019; Xiao et al. 2022
*Hibiscus mutabilis* L. (Fu rong ye)	Tiliroside	Oral	Suppressed viral growth of various IAV strains, targets viral ribonucleoprotein, and reduces expression of cytokines and chemokines.	Xiao et al. 2023
*Alstonia scholaris*	Scholaricine, picrinine, 19‐epischolaricine, and vallesamine	Intravenous	Activates β2 adrenergic receptor and inhibition of NF‐κB expression. Suppresses H1N1 virus replication and extends survival time of mice with a minimum dose of 12.5 mg/kg.	Zhao et al. 2021
*Punica granatum*	Ellagic acid, ellagitannins	Oral	Prevent synthesis of various inflammatory indicators and reduce promoter inhibition. Reduces PGE2 synthesis, hinders NF‐κB activation, downregulates MAPK, and suppresses COX‐2 and mPGES‐1 expression.	González‐Sarrías et al. 2010; H. Yu et al. 2020; Zarfeshany, Asgary, and Javanmard 2014
*Epimedium koreanum* Nakai	Quercetin, icariin, epimedosides, hyperoside, icariside II, epimedin, and chlorogenic acid	Oral	Antimicrobial, anti‐inflammatory, and antioxidant activity. Exhibits HO‐1 expression, reduces iNOS and COX‐2 expression, suppresses NO, IL‐1β, and IL‐6 production.	Qian et al. 2024; Yasmin et al. 2020 Cho et al. 2012
*Scutellaria baicelensis* Georgi	Baicalin	—	Minimize miR‐146a expression, suppresses CXCL1, IL‐8, and NF‐κB. Has effect on both H1N1 and H3N2 viruses.	Kivihall et al. 2019; R. Li and Wang 2019; Mousa 2017
*Paeonia lactiflora* Pallas	Paeoniflorin	—	Interferes with viral binding to and penetration into host cells, lowers synthesis of inflammatory media, modifies immune cell activation and function, and repairs aberrant signal pathways.	Mousa 2017; L. Zhang and Wei 2020
*Melaleuca alternifolia*	Terpinen‐4‐ol and α‐terpineol	—	Inhibit entry and fusion of influenza virus, disrupt acidification process of intralysosomal compartment.	Madia et al. 2022
*Curcuma longa*	Bisabolane‐type sesquiterpenoids	—	Inhibits replication of H1N1, suppresses production of pro‐inflammatory mediators.	Ti et al. 2021
*Curcuma zedoaria*	Germacrone	Intraperitoneal	100 mg/kg of germacrone causes reduction in antiviral proteins expressions such as RIG‐I, IFNs, OAS, IRF3/7, MX, and EIF2AK2/PKR, heightens TAP1 expression, blocks TAK1 phosphorylation, and suppresses NF‐κB signaling.	L. Li et al. 2020
Eucalyptus	Eucalyptol	—	Lowers cytokine levels and suppresses inflammatory responses in pulmonary tissues.	Ait‐Ouazzou et al. 2011
Shufeng Jiedu capsule (SFJDC)	Saikosaponins, resveratrol, emodin, forsythoside A, glycyrrhizin, indirubin, vitexin, quercetin	Intragastric	Downregulates IL‐1β and IL‐18 levels in serum and BAL in vivo, and decreased the expression of NLPR3‐associated components with a dose of 0.6 g/kg/day.	Ji et al. 2020
Lianhuaqingwen (LHQW)	Forsythoside A, forsythoside I, rhein, glycyrrhizin, amygdalin, neochlorogenic acid, rutin, prunasin, quercetin, forsythoside E, hyperoside, and kaempferol	Oral	Inhibits influenza replication and inflammatory cytokines in both in vitro and in vivo experiments. Dosage up to 1300 mg reduced inflammatory cytokines such as TNF‐α and IL‐6.	Ding et al. 2017; X. Shen and Yin 2021

### 2.4 Pneumonia

Pneumonia is classified as a sudden inflammation of the lower airway tract and stands as one of the foremost contributors to global mortality (Reynolds et al. 2010). According to reports, *Chlamydia pneumoniae* can infect astrocytes, microglia, and neurones as well as pass across the blood–brain barrier through infected monocytes or the olfactory pathway. Moreover, it can promote the persistence of chronic infection and suppress neuronal death (Wadhwa et al. 2020). Causes of pneumonia include bacteria, viruses, and fungi. The most common bacteria that causes pneumonia is *Streptococcus pneumoniae*, while common viruses that cause pneumonia in adults are influenza virus or rhinovirus (NIH 2022). In children, respiratory syncytial virus (RSV) is the main causative agent of viral pneumonia, a pathogen that mainly infects the lower respiratory system and cause severe lung inflammation such as pneumonia, bronchiolitis especially in children younger than 1 year of age (L. L. Lin et al. 2016; NIH 2022). Based on data by WHO, approximately 15% of deaths among children under the age of 5 in 2017 worldwide were attributed to pneumonia (Kumbhar et al. 2023). On the other hand, *Pneumocystis jirovecii*, *Aspergillus*, and *Cryptococcus* are examples of fungi that cause pneumonia, particularly in individuals with compromised immune systems (Z. Li, Lu, and Meng 2019; NIH 2022). However, in adults, pneumonia can also arise from nonpathogenic factors like smoking. Based on the appearance of the chest radiograph, adults with pneumonia can be roughly categorized into three types: lobar pneumonia, bronchopneumonia, and pneumonia exhibiting an interstitial pattern. Lobar pneumonia is most frequently linked to hospital‐acquired infections, community‐acquired infections, and an interstitial pattern associated with the so‐called atypical pneumonias, which can be brought on by viruses or microorganisms like *Mycoplasma pneumoniae* (Reynolds et al. 2010).

Existing vaccines and medications for preventing and treating viral pneumonia are hindered by issues such as neurotoxicity and limited efficacy. Therefore, there is a requirement to identify novel medications for viral pneumonia with reduced toxicity (Jiao et al. 2023). In a clinical study involving 155 patients suffering from acute lower respiratory infections such as acute bronchiolitis, bronchial asthma, or pneumonia, they were targeted to be assessed with different herbal products and evaluate the prevalence of various herb remedies. A total of 59.3% of people were reported to use herbal products for treatment. This shows that the usage of herbal treatment for acute lower respiratory diseases is common in Saudi Arabia, but their safety and efficacy need to be further assessed (Alharbi et al. 2018).

In viral pneumonia, the outer layer of membrane proteins in mitochondria will activate NLRP3 inflammasome, leading to further activation of signaling pathways and oligomerizations (Kanneganti et al. 2006). Moreover, PB1‐F2 NSP of viral RNA is involved in the activation of inflammasome, which is able to further activate IL‐1β by clustering phagosome (McAuley et al. 2013). Apart from inflammasomes and cytokines, induction of type I and type III IFN also occurs (van Kempen et al. 2015). While for bacterial pneumonia, various inflammasomes are activated due to the host's natural immunological reaction. The main inflammasome activated is also NLRP3 (Ravi Kumar et al. 2018). Pneumolysin‐activated NLRP3 provides protective immunity toward pneumococci via regulating lung barrier integrity and limiting the growth of bacteria *Klebsiella pneumoniae* (Witzenrath et al. 2011). Genomic DNA from *S*. *pneumoniae* activates AIM2 inflammasome, subsequently triggering caspase‐1 activation as well as IL‐1β and IL‐18 maturations in macrophages (Fang et al. 2011). Besides, NLRP12 is activated and its effect is moderated via IL‐17A or CXCL‐1 (Cai et al. 2016). NLRC4 inflammasomes such as NLRC4, caspase‐1, ASC, and NLR family apoptosis inhibitory protein (NAIP) are expressed (Franchi et al. 2006).

#### 2.4.1 Viral Pneumonia

Curcumin is classified as an active phenolic compound that can be extracted from *C*. *longa* is a good candidate against cancer, suppresses inflammatory reactions, and defends the central nervous system which is neuroprotective. However, it is poorly soluble and quickly removed from the plasma (Y. Han et al. 2022). In a study, a novel hydrophilic formulation of curcumin (CDC) was formulated to fix the limitations in physiological chemistry and completely utilize the potentiality of curcumin for pulmonary infections. The administration of CDC to deliver curcumin resulted in lowering in lung damage, inflammation, oxidative stress, blood and lung bacterial presence, and death. Curcumin can decrease hypoxia‐inducible factor 1/2α, *Klebsiella* hemolysin gene expression, nucleotide‐binding domain, TNF‐α, leucine‐rich‐containing family, IFN‐β, pyrin domain–containing‐3, and NF‐κB. This suggests that the primary mechanism attained by the CDC able to diminish pneumonia severity is modulation of the hypoxia signaling pathways and inflammasome complex. Consequently, curcumin also shows promise for reducing inflammation in pneumonia and maybe other inflammatory pulmonary conditions, underscoring the need of enhancing the physicochemical characteristics of active natural products to maximize their therapeutic use (B. Zhang et al. 2019). Besides, animal trials have demonstrated that high‐dosage curcumin has powerful antibacterial activity (Gunes et al. 2016). The impact of curcumin on IAV replication was investigated in both the mouse model and the A549 cell line. In vitro experiments revealed that curcumin effectively inhibited IAV, and in a mouse model, it mitigated the severity of the disease after IAV infection. Additionally, the study also demonstrated that curcumin induced the expression of HO‐1 in vivo, providing protection against IAV‐induced lung tissue damage. Moreover, following an IAV infection, curcumin had a regulatory impact. This substance suppresses the generation of local inflammatory cytokines, which modifies the immune response (S. Han et al. 2018). Curcumin and *C*. *longa* extract have been shown to be safe when administered in doses ranging from 120 to 1500 mg for 4 to 36 weeks. According to FAO/WHO and EFSA, the acceptable daily intake is 0–3 mg/kg, and the US FDA has approved curcumin as a botanical. Clinical trials indicate that the maximum efficacious dose is 4–8 g/day, with doses up to 12 g/day being well‐tolerated. Studies report no increase in adverse events and no significant subchronic toxicity, mutagenic, or teratogenic effects. Animal studies on demethyl curcumin (DC) show an acute oral LD50 of > 5000 mg/kg and an acute dermal LD50 of > 2000 mg/kg, with no adverse effects observed. Curcumin‐loaded NPs were found to be nontoxic at 2000 mg/kg and safe for long‐term use at therapeutic doses. Overall, curcumin demonstrates broad‐spectrum safety across various doses and formulations (Zeng et al. 2022). In summary, curcumin from *C*. *longa* shows promise against cancer, inflammation, and neuroprotection but suffers from poor solubility. A hydrophilic formulation (CDC) addresses this, reducing lung damage and inflammation in pneumonia. It also exhibits antibacterial activity and inhibits influenza A virus replication while being safe in various doses and formulations, with no significant adverse effects observed.

Medicinal mushrooms have also been used and explored for thousands of years. *Ganoderma lucidum*, or widely known as ling zhi, is a popular edible mushroom that has shown remarkable anti‐inflammatory properties. Its constituents, including polysaccharides and triterpenes, have been proven to be the major substituents that reduce inflammatory responses (J. Xu et al. 2021). *G*. *lucidum* polysaccharides (GLP), such as β‐glucan, have been demonstrated to decrease lung injury induced by LPS in mice. It prevents pneumonia by its anti‐inflammatory effects. GLP decreases inflammation and guards against pneumonia by decreasing the C‐caspase 3/caspase 3 ratio and increasing Bcl‐2/Bax ratio, while also regulating cellular processes such as suppressing NRP1 expression, inducing cellular autophagy, and preventing cell apoptosis. Expression levels of pulmonary IL‐1β, IL‐6, TNF‐α, and Saa3 were suppressed at GLP dosages of 25 and 50 mg/kg (X. Zhang et al. 2022). Moreover, acetone extract derived from *G*. *lucidum* had demonstrated significant antibacterial efficacy, particularly against *K*. *pneumoniae* (Quereshi, Pandey, and Sandhu 2010). Another constituent that was on research was *G*. *lucidum* sterols (GLS). Experiments on RAW264.7 cell line showed that GLS suppressed inflammation and reduced pro‐inflammatory mediator mRNA expression including NO, TNF‐α, IL‐1β, and IL‐6. Furthermore, it also stopped p38 from becoming phosphorylated in MAPK pathways and prevented IκB‐α phosphorylation and degradation, while also blocking NF‐κB p65 phosphorylation in NF‐κB pathways (J. Xu et al. 2021). These in vitro and in vivo experiments on *G*. *lucidum* have demonstrated its role as a natural anti‐inflammatory agent for prevention and treatment of inflammatory conditions. In summary, *G*. *lucidum* possesses potent anti‐inflammatory properties due to its polysaccharides, triterpenes, and sterols. These compounds reduce inflammation, prevent pneumonia, and exhibit antibacterial efficacy, making a promising natural agent for inflammatory conditions. However, further clinical studies would be needed to determine its adverse reactions and safety profile in humans.

*Tamarix chinensis* Lour. is employed in TCM to treat measles or cases of measles complicated by pneumonia. Its polysaccharides are found to be complement inhibitors. As excessive activation of the complement system is associated with pneumonia induced by H1N1 in mice, the polysaccharides were deemed beneficial in preventing pneumonia (Jiao et al. 2022; Zhu et al. 2018). Two polysaccharides substituted with quercetin, MBAP‐1 and MBAP‐2, were extracted from its crude polysaccharide MBAP90. Both compounds exhibited strong anticomplement and antioxidant properties in vitro, with MBAP‐1 exhibiting greater potency. It is thought that their potential synergistic action between active flavonoid and polysaccharide components could provide beneficial effects against viral pneumonia. (Jiao et al. 2023) On the other hand, MBAP90 was found to lengthen the survival of H1N1‐ALI mice by 50%, especially with a higher dose of 400 mg/kg. Not only that, but acute lung injury was also decreased in mice administered with 200 and 400 mg/kg of MBAP90. Another polysaccharide was isolated from MBAP90, MBAP‐3, which exhibits robust in vitro anticomplement activity at a low dose of 50 mg/kg. It has been proposed that MBAP90 functions by controlling the ratio of pro‐ to anti‐inflammatory cytokines and inhibiting the excessive activation of the complement system (Jiao et al. 2023). In summary, *T*. *chinensis* Lour. polysaccharides inhibit complement activity, potentially preventing H1N1‐induced pneumonia. Extracted compounds like MBAP‐1 and MBAP‐2 show strong anticomplement and antioxidant properties.

Besides, the findings align with earlier research that showed the antiviral properties of *E*. *globulus* and *C*. *zeylanicum* EOs on H1N1 and HSV1 (Astani, Reichling, and Schnitzler 2010; Vimalanathan and Hudson n.d.). For instance, 1,8‐cineole and β‐caryophyllene, two components of eucalyptus EO, directly render virus particles inert, demonstrating anti‐HSV1 activity. One of the main molecular mechanisms underlying the antiviral impact that inhibits viral infection is direct inactivation, or the direct interaction of monoterpenes with free viruses—more especially, regarding the viral protein implicated in the process of host cell entry and penetration (Mieres‐Castro et al. 2021). Additionally, they may impede structures of the virion envelope necessary for the virus to infect host cells (Astani, Reichling, and Schnitzler 2010, 2011). Antiviral drugs that are often used, such as ganciclovir and acyclovir, to inhibit DNA polymerases. A prior published study demonstrated the efficacy of a blend of rosemary, orange, cinnamon, clove, and eucalyptus oil (Wu et al. 2010). However, the blend's antibacterial properties were not examined. According to Chung and Huh's (2015) study, AB1 has demonstrated efficacy against various viruses and bacteria, for instance, H1N1 virus, *S*. *pneumoniae* as well as *S*. *aureus*, which are the two bacteria that cause post‐influenza pneumonia (Chung and Huh 2015). In summary, *E*. *globulus* and *C*. *zeylanicum* EOs have antiviral properties against H1N1 and HSV1, deactivating virus particles directly. A blend of oils, including rosemary, orange, cinnamon, clove, and eucalyptus, has shown efficacy against viruses and bacteria, including those causing post‐influenza pneumonia.

#### 2.4.2 Bacterial Pneumonia

*Carum carvi*, often known as caraway, is a traditional remedy for pneumonia, dyspepsia, and as an appetizer, carminative, and galactagogue. Caraway is used to make fixed oil, EO, and various crucial chemicals for factorial uses. The main components of caraway oil are composed of carvone and limonene. D‐Limonene exhibits mechanisms of binding to P13K and NF‐κB p65 and inhibiting phosphorylation and PI3K/Akt/IKK‐α/NF‐κB p65 signaling pathway expression (F. Yang et al. 2021). Hence, multiple pro‐inflammatory genes expression will be prevented such as cytokines, chemokines, and inflammasome regulation (T. Liu et al. 2017). Due to insufficient data, caraway oil is not advised for use in adults under the age of 18, although it can be topically applied to children or infants as a carminative and anti‐colic agent. Caraway oil's anti‐aflatoxigenic, antioxidant, and antibacterial properties could be used as medicinal herbs against pneumonia. However, there is no strong scientific proof to support that caraway is able to treat pneumonia (Mahboubi 2019). There was research that delved into examining the effects of EO of *C*. *carvi L*. seed (CEO) on methicillin‐resistant *Staphylococcus aureus* (MRSA) and investigating its potential mechanism of action. The principal chemical constituents of CEO, identified through GC–MS, were carvone and limonene. Notably, CEO exhibited substantial inhibitory effects on both planktonic bacteria and biofilm growth in MRSA cells. To elucidate the interaction mechanism between MRSA and CEO, untargeted metabolomics utilizing GC‐Q‐TOF‐MS was employed. The analysis revealed 63 distinct metabolites with fold change values exceeding 1.5 or falling below 1.5, statistical significance indicated by *p* values less than 0.05, and variable importance in projection (VIP) scores surpassing 1 which indicating a significant impact on amino acid metabolism in MRSA due to CEO. In summary, CEO demonstrates robust antimicrobial properties, highlighting its prospective applicability within the realms of both pharmaceuticals and food science (C. Liu et al. 2023). In summary, *C*. *carvi* has been traditionally used for various purposes including treating pneumonia. Its main components, carvone and limonene, exhibit anti‐inflammatory properties by inhibiting pro‐inflammatory gene expression.

Many plants are used in Assamese folk medicine to cure pneumonia, but scientific evidence supporting the plants' bioactivity is needed before these traditional uses may be included into the mainstream medical system. *Mucuna pruriens* chloroform extract and *Xanthium strumanium* petroleum ether extract demonstrated significant bactericidal properties against *S*. *pneumoniae* (15.33 mm) and *S*. *aureus* (15 mm), respectively, with larger zones of inhibition than the control, chloramphenicol. Flavonoids, sesquiterpenoids, coumarins, steroids, phenylpropenoids, lignanoids, glycosides, anthraquinones, and naphthoquinones are among the compounds that make up xanthium. Significant medical benefits for some of these substances, including diuretic, anthelmintic, antifungal, anti‐inflammatory, antidiabetic, and anticancer effects, have been demonstrated (J. Zhang et al. 2022). The bulk of the investigated medicinal plants, such as *X*. *strumanium* and *M*. *pruriens*, is used in traditional medicine, and the current study offers scientific support for their use. Results regarding its toxicity indicates that Xanthium plant crude extracts are generally harmless for utilization (Linh et al. 2021). These plants may also be a future source of alternative antibacterial medications (Kalita and Kalita 2014). In vitro study of extracts in methanol of leaves of *Xanthium strumarium* L. was assessed for the antioxidant and antimicrobial properties against both methicillin‐susceptible and MRSA. The IC50 values for antioxidant and DPPH‐scavenging capacity were determined to be 0.02 and 0.09 mg/mL, respectively. The extract showed effects on both MRSA and methicillin‐sensitive *Staphylococcus aureus* (MSSA) with a stronger antibacterial efficacy against methicillin‐susceptible *S*. *aureus* species. The observed antioxidant and antibacterial properties of the methanol extract corroborate the traditional medicinal use of this plant across various cultures (Rad et al. 2013). In the case of *M*. *pruriens*, the administration of *M*. *pruriens* at a dosage of 10 mg/kg and diclofenac potassium at 10 mg/kg resulted in a reduction in inflammation by 9.8% and 62.0%, respectively. A further reduction was shown by increasing the dosage of *M*. *pruriens* to 50 mg/kg, with rates of 47.80% and 38.80% for acute inflammation and chronic inflammation, respectively. These findings indicate that the crude extracts from *M*. *pruriens* seeds exhibit significant anti‐inflammatory activity in rats. Moreover, the seeds demonstrated high antioxidant activities. Additionally, extracts from *M*. *pruriens* seeds displayed robust antibacterial activities against various tested organisms, including *S*. *aureus*, *Proteus mirabilis*, *K*. *pneumoniae*, *Escherichia coli*, *and Pseudomonas aeruginosa*. The seed extract stopped bacterial growth at a concentration of 12.5 mg/mL for most organisms, except for *K*. *pneumoniae*, where a concentration of 25.00 mg/mL was required. This suggests that seeds of *M*. *pruriens* have potential to be utilized as a remedy for antioxidant herbs, infections as well as inflammation in herbal medical practices (Uchegbu et al. 2016). However, there are no clinical trials found for the plant.

Alternative chemicals with antibacterial action are highly needed since harmful bacteria, particularly *K*. *pneumoniae*, are becoming increasingly resistant to treatments (Imran, Jha, Hasan, Insaf, et al. 2022). Here, *Lycopersicon esculentum* L. was used to isolate lycopene. One of the primary dietary carotenoids found in tomatoes and certain other fruits is lycopene. When *C*. *trachomatis and C*. *pneumoniae* infections occur in alveolar macrophages, it has a potent inhibitory impact (Zigangirova et al. 2017). To evaluate the binding processes of lycopene to a few key proteins of *K*. *pneumoniae*, a molecular docking technique was utilized. Antibacterial efficacy of lycopene was tested against *K*. *pneumoniae*, where it was observed to induce membrane fluidization, enhancing the pliability of biological membranes, including those found in bacterial cell walls. In vivo experiment shows inflammation and congestion had reduced in mice treated with lycopene in bilosomes. It was shown that bilosomes nanovesicles hold promise to improve oral delivery and effectiveness of highly hydrophobic components in vivo (Binsuwaidan et al. 2022). In a research paper, findings indicate that lycopene exhibits a potent inhibitory effect on infections induced by *C*. *pneumoniae and C*. *trachomatis* in alveolar macrophages via application of electron microscopy and immunofluorescence analysis. Notably, lycopene treatment was observed to impede chlamydial developmental cycle intracellular phase, leading to an obvious reduction in the production of infectious progeny. For toxicity assessment, long‐term treatment with the highest dose (1000 mg/kg) of tomato leaves extract could manifest lower toxic effects, as indicated by significant increases in urea levels and total serum proteins at this dose compared to the control. However, microscopic examination revealed no remarkable pathological changes in the organs of treated male and female rats. These findings suggest that a single dose of tomato leaves extract is relatively nontoxic at 5000 mg/kg body weight. Prolonged use of lower doses (250 and 500 mg/kg) is recommended for oral administration, while the highest dose (1000 mg/kg) should be avoided (Nguenang, Ntyam, and Kuete 2020).

The anti‐chlamydial efficacy of lycopene was further supported by clinical evidence. Volunteers who received oral ingestion of 7 mg of lycopene for a month showed a substantial reduction in IgG antibodies against *C*. *pneumoniae* in their serum. These findings prove that lycopene has the potential to combating chlamydial infections and prompt the need for additional studies to delve deeper into its anti‐chlamydial activity and its possible impact on *C*. *pneumoniae*, particularly related to mechanisms associated with atherosclerosis (Zigangirova et al. 2017).

Purple sweet potato (*Ipomoea batatas*) contains abundant anthocyanins, which possess extensive anti‐inflammatory and antioxidant properties. Purple sweet potato anthocyanins (PSPAs) extracted were tested to contain 98.7% of delphinidin 3‐sambubioside. In alveolar macrophages infected with *K*. *pneumoniae*, PSPAs notably inhibited pyroptosis and limited the activation of NLRP3 inflammasome. Not only that, PSPAs with a dosage of 30 mg/kg increased survival rates in 50% of mice infected with *K*. *pneumoniae*. Bacterial CFUs in the lungs of the PSPAs‐treated mice were also lower. Furthermore, mice treated with PSPAs experienced decreased lung injury and inflammatory responses, alongside reversal of lung abscess and pulmonary consolidation. PSPAs stimulated mitophagy via Nrf2 signaling pathway, which enhances mitochondrial membrane potential and inhibits NLRP3 inflammasome activation. Hence, PSPAs have the potential to be further explored to treat *K*. *pneumoniae* infection (Dong et al. 2021). Another study on *I*. *batatas* demonstrated that oral administration of PSPY in mice up to 5 g/kg body weight did not result in acute toxicity. Additionally, a dosage of up to 40 g/kg body weight did not lead to subchronic toxicity (Khairani et al. 2022). In summary, PSPAs extracted from *I*. *batatas* demonstrate potent anti‐inflammatory and antioxidant effects. Table 3 shows a summary of all plants mentioned in this segment.

**TABLE 3:** Medicinal plants with their respective active constituents and its properties to treat viral and bacterial pneumonia.

Plant	Active constituent	Method of delivery	Properties	References
Viral pneumonia				
*Curcuma longa*	Curcumin	Oral	Decreases the expression of the *Klebsiella* hemolysin gene, IFN‐β, TNF‐α, nucleotide‐binding domain, pyrin domain–containing‐3, leucine‐rich‐containing family, hypoxia‐inducible factor 1/2α, and NF‐κB, and induces the expression of HO‐1 in vivo. No increase in adverse reaction and subchronic toxicity. Acceptable daily intake: 0–3 mg/kg. Highest tolerated dose in human: Up to 12 g/day.	Zeng et al. 2022; B. Zhang et al. 2019; S. Han et al. 2018
*Ganoderma lucidum*	Polysaccharides and triterpenes	Intraperitoneal and intragastric	Reduces LPS‐induced lung injury in mice, decreases the C‐caspase 3/caspase 3 ratio and increasing Bcl‐2/Bax ratio, suppresses NRP1 expression, inducing cellular autophagy and prevent cell apoptosis, suppresses inflammation and lowers mRNA levels of pro‐inflammatory mediators such as NO, TNF‐α, IL‐1β, and IL‐6 at GLP dosages of 25 and 50 mg/kg.	J. Xu et al. 2021; X. Zhang et al. 2022
*Tamarix chinensis*	Polysaccharides	Oral	Exhibits strong anticomplement and antioxidant properties in vitro, inhibits the over‐activation of complement system, and balances pro‐inflammatory and anti‐inflammatory cytokine. MBAP90 was found to lengthen the survival of H1N1‐ALI mice by 50%, especially with a higher dose of 400 mg/kg.	Jiao et al. 2023
*E*. *globulus* and *C*. *zeylanicum* EOs	1,8‐Cineole and β‐caryophyllene		Render virus particles inactive.	Mieres‐Castro et al. 2021
Bacterial pneumonia				
*Carum carvi*	Carvone and limonene	Topically	Inhibits phosphorylation and PI3K/Akt/IKK‐α/NF‐κB p65 signaling pathway expression, prevents expression of multiple pro‐inflammatory genes such as cytokines, chemokines, and inflammasome regulation.	C. Liu et al. 2023; T. Liu et al. 2017; F. Yang et al. 2021
*Mucuna pruriens* chloroform extract and *Xanthium strumanium* petroleum ether extract	Flavonoids, sesquiterpenoids, coumarins, steroids, phenylpropenoids, lignanoids, glycosides, anthraquinones, naphthoquinones	*—*	*Mucuna pruriens* Anti‐inflammatory activity in rats and antibacterial activities against various tested organisms, including *S*. *aureus*, *Proteus mirabilis*, *K*. *pneumoniae*, *Escherichia coli*, *and Pseudomonas aeruginosa*. *Xanthium strumanium* Strong antibacterial efficacy against methicillin‐susceptible *S*. *aureus* species.	Rad et al. 2013; Uchegbu et al. 2016
*Lycopersicon esculentum* L.	Lycopene	Oral	Induce membrane fluidization, enhancing the pliability of biological membranes, and exhibits a potent inhibitory effect on infections caused by *C*. *trachomatis and C*. *pneumoniae* in alveolar macrophages. Minimal toxic dose: 1000 mg/kg.	Binsuwaidan et al. 2022; Nguenang, Ntyam, and Kuete 2020
*Ipomoea batatas*	Anthocyanins	Intraperitoneal	Inhibits pyroptosis and limits the activation of NLRP3 inflammasome, stimulates mitophagy via Nrf2 signaling pathway, enhances mitochondrial membrane potential, and inhibits NLRP3 inflammasome activation.	Dong et al. 2021

### 2.5 Tuberculosis

Globally, TB remains a serious health concern, contributing significantly to mortality rates across the world, even with its history that dates back a few thousand years ago (CDCTB 2023; Khatak et al. 2020; Natarajan et al. 2020). It is caused by *Mycobacterium tuberculosis* or *Mycobacterium africanum*, which are closely related. A smaller proportion of cases are attributed to zoonotic species belonging to the *M*. *tuberculosis* complex, including *M*. *bovis* or *M*. *caprae* (Reed et al. 2009). Notably, *M*. *tuberculosis* lacks a known environmental reservoir and humans serve as its exclusive reservoir, permitting it to acquire resistance to antibacterial medications gradually (Furin, Cox, and Pai 2019). *M*. *tuberculosis* had a major contribution to antimicrobial resistance, allowing it to emerge as the primary infectious cause of global mortality with 1.5 million deaths every year (WHO, n.d.). The treatment of TB requires administering several drug combinations for an extended period, usually 6 months with four different antibiotics (Dua et al. 2018; WHO n.d.).

In the lungs, blood macrophages that come into contact with the bacterial infection experience a respiratory burst, producing large quantities of ROS. Production of ROS leads to lipid peroxidation, elevated intracellular calcium levels, and eventually damaging the host DNA (Dua et al. 2018). In latent TB infection (LTBI), *M*. *tuberculosis* remains metabolically active and continues to replicate within host tissues, despite the absence of any clinical signs or symptoms of the disease. Evidence suggests that in LTBI, *M*. *tuberculosis* can inhabit various organs, tissues, and cell types, even those that are not linked to the primary infection site and may not show the typical granulomatous lesions (Delogu, Sali, and Fadda 2013). Severe immunosuppression accounts for only 10% of active TB cases, with the majority being ascribed to underlying health conditions, an inflammatory environment, and an uncertain genetic predisposition (Cardona 2018). TB activates and produces proinflammatory cytokines and inflammasomes to boost the host immunity. TB activates IFN‐γ which is secreted by macrophages and T cells to improve the bactericidal strength (Wawrocki and Druszczynska 2017). The antimicrobial abilities of phagocytes stimulated by IFN‐γ are enhanced by IL‐18 and IL‐1β, which are proinflammatory cytokines processed by caspase‐1 (Saiga et al. 2015). In addition to IFN‐γ, multiple cytokines, for instance, IL‐4, IL‐5, IL‐13, IL‐17, and TNF‐α, are produced (Wawrocki and Druszczynska 2017).

**FIGURE 4:**
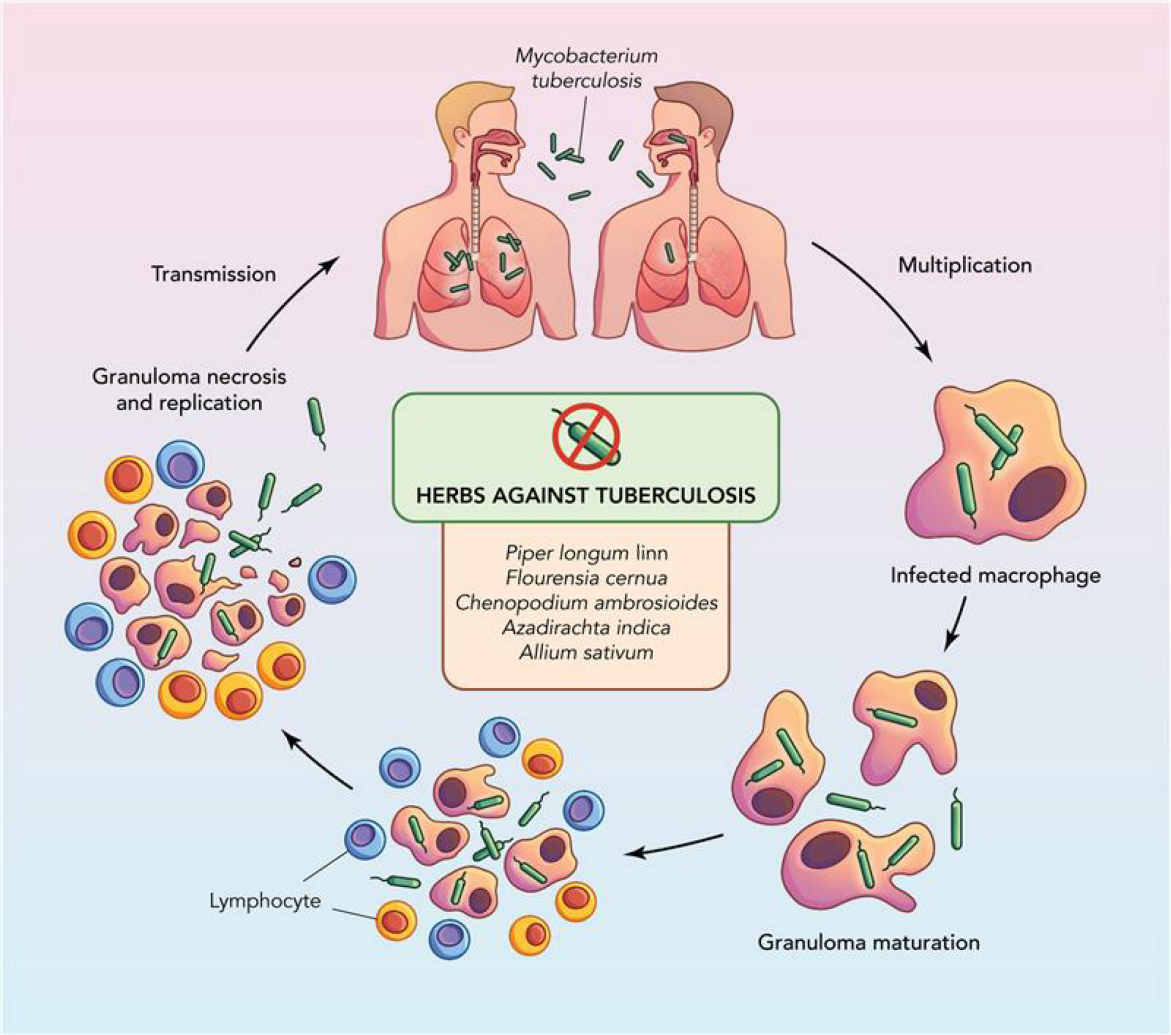
Mechanism of action of herbs that treat tuberculosis.

In South Asia and Africa, traditional medicines offer a wealth of medicinal plants and compounds derived from plants that serve as a viable option for treating various diseases in humans. While substantial research exists on the therapeutic potential of naturally derived compounds for numerous ailments, there has been a significant lack of investigation into their effectiveness for TB. Phytoproducts, derived from plants, are now under investigation as potential anti‐mycobacterial agents since the current treatments face limitations due to their low effectiveness, high toxicity to the liver, extended treatment time, patient nonadherence, and the emergence of multidrug‐resistant (MDR) and extensively drug‐resistant (XDR) TB (Dua et al. 2018; Gautam et al. 2023). These compounds are under investigation for their capacity to diminish bacterial load or regulate the immune system, with the goal of minimizing adverse effects during TB treatment (Gautam et al. 2023). Figure 4 schematically shows the various mechanisms of herbs that are used in the management of TB.

Piperlongumine (PL) and piperine are bioactive alkaloids and medicinal compounds that can be derived from the roots of *P*. *longum* Linn. PL exhibited the capacity to inhibit inflammatory mediators and prevent lymphocyte function‐associated antigen 1 from adhering to MH‐S cells following trehalose 6,6′‐dimycolate (TDM) activation. Furthermore, it has demonstrated the ability to augment the phagocytic clearance of *M*. *tuberculosis* by macrophages. PL profoundly alters the Syk‐ERK‐lectin receptor (Mincle)‐inducible Ca^2+^‐dependent macrophage‐inducible signaling pathway in TDM‐stimulated MH‐S cells. The oral administration of PL has proven efficacious in suppressing the growth of lung granulomas and ameliorating responses to inflammation in a murine granuloma model induced by TDM (Lu et al. 2021). As for piperine, it has a synergic interactions with the combination of the Rifampin in killing *M*. *tuberculosis* (Murase et al. 2019). In short, PL from *P*. *longum* Linn roots inhibits inflammatory mediators, enhances macrophage phagocytic activity against *M*. *tuberculosis*, and modulates macrophage‐inducible signaling pathways.

Besides, *Flourensia cernua*, commonly referred to as *hojasen*, is recognized as a shrub with widespread distribution in northern Mexico and the southern United States. Notably, *F*. *cernua* is a plant native to the Coahuila area in Mexico. Its leaves and blossoms are readily available in local markets across Mexico and are commonly used to create infusions for traditional medicinal uses (Linares Braham, Palomo‐Ligas, and Nery‐Flores 2023). *F*. *cernua* is rich in flavonoid‐type compounds (Aranda‐Ledesma et al. 2022). Several investigations have demonstrated that extracts from *F*. *cernua* consist of bioactive compounds that function as potent in vitro antioxidants, effectively mitigating the harmful effects of free radicals. Additionally, these extracts exhibit antifungal properties against phytopathogenic fungi and display notable antibacterial effectiveness against *M*. *tuberculosis* (Linares Braham, Palomo‐Ligas, and Nery‐Flores 2023). Notably, the extracts not only inhibit the growth but also induce the death of *M*. *tuberculosis*. Signs of toxicity in *F*. *cernua* include hepatotoxicity, specifically affecting the liver when the leaves are used (Alonso‐Castro et al. 2017). In specific studies, the hexane extract displayed a minimal inhibitory concentration (MIC) of 50 μg/mL for sensitive *M*. *tuberculosis* strains and 25 μg/mL for resistant strains. Additionally, the acetone extract showed activity against the CIBIN:UMF:15:99 strain with an MIC of 100 μg/mL (Molina‐Salinas et al. 2006). Moreover, the material extracted using hexane from the decoction exhibited 55.17% and 92.62% antituberculosis activity against sensitive and resistant strains, respectively (Molina‐Salinas et al. 2011). In short, *Flourensia cernua* is rich in flavonoids, display antioxidant properties, and combat fungal infections and *M*. *tuberculosis*. However, due to insufficient information, further research is required to determine the optimal route of delivery.

In Ghanaian ethnomedicine, *Chenopodium ambrosioides* leaves are frequently employed for addressing chronic coughs and other associated symptoms affecting the lower respiratory airway (Nguta et al. 2016). *C*. *ambrosioides* L. belongs to the Amaranthaceae family. It has demonstrated potential activity against MDR strains of *M*. *tuberculosis*, with its bioactive constituents such as phenolics, flavonoids, saponins, ecdysteroids, and triterpenoids (Nguta et al. 2015). Acute toxicity tests revealed that the mean lethal dose (LD50) for both aqueous and methanol extracts of *C*. *ambrosioides* is greater than 2000 mg/kg. Under acute conditions, a single oral administration of these extracts effectively lowered fasting blood glucose levels in rats (Kasali et al. 2022). For in vitro study of *C*. *ambrosioides* L., the ethyl acetate fraction demonstrated significant antimicrobial activity, inhibiting a diverse range of microorganisms, and exhibiting the lowest minimum inhibitory concentration (MIC) values against *S*. *aureus* and *Enterococcus faecalis* (MIC = 0.42 mg/mL), *P*. *aeruginosa* (MIC = 34.37 mg/mL), *Paenibacillus thiaminolyticus* (MIC = 4.29 mg/mL), and *Paenibacillus apiarus* (MIC = 4.2 mg/mL). Furthermore, the chloroform fraction demonstrates significant antimycobacterial activity against *M*. *tuberculosis*, *M*. *smegmatis*, and *M*. *avium*, with MIC values ranging from 156.25 to 625 μg/mL. Hence, it can be a potential treatment of TB (Jesus et al. 2018). In summary, *C*. *ambrosioides* leaves, used in Ghanaian ethnomedicine for chronic coughs, show promise against drug‐resistant strains of *M*. *tuberculosis* due to their bioactive compounds.

The bark of *Azadirachta indica* A. Juss, or neem, has been traditionally employed to address conditions such as coughing up blood and phlegm (Indriana and Salman 2020). As a member of the Meliaceae family, neem is recognized for its health‐promoting effects attributed to its rich antioxidant content (Alzohairy 2016). *Azadirachta indica* contains nimbin, nimbolide, nimbidin, and limonoids, which play a role in disease management by modulating genetic pathways and exhibiting other activities. Notably, quercetin and ß‐sitosterol, classified as polyphenolic flavonoids in its leaves, have demonstrated antifungal and antibacterial activities (Govindachari et al. 1998). The mechanism of action includes the in vitro inhibition of *M*. *tuberculosis* growth by the ethanol extract of neem bark. Toxicity testing, acute toxicity assessment, and histopathological observations were conducted in mice. Tablet preparations of neem bark ethanol extract were formulated through wet granulation, and their potential as an antituberculosis compound was assessed in animal models infected with *M*. *tuberculosis* H37Rv. Changes in biochemical parameters due to toxicity can significantly impact animal organ function. Hence, caution should be exercised when considering the ethanolic extract of *A*. *indica* stem bark as an oral remedy, especially at doses ranging from 50 to 300 mg/kg body weight (Ashafa, Orekoya, and Yakubu 2012). In the flower part of neem, ethanol extracts exhibited antitubercular activity, with the ethanolic extract's MIC found to be 25 μg/mL compared to the standard rifampicin with an MIC of 12.5 μg/mL (Nagasree et al. 2022). In vivo efficacy of neem bark extract tablets in curing TB in experimental animals infected with *M*. *tuberculosis* H37RV was demonstrated. Animals receiving a dose of two tablets (50 mg/tablet) three times a day for 6 weeks showed a reduction in *M*. *tuberculosis* bacteria from +3 to negative (Indriana and Salman 2020). Results from studies on human monocytes infected with *M*. *tuberculosis* indicated that neem extract suppressed TNF‐α and MTB Ag85B proteins in a dose‐dependent manner. Following infection with *M*. *tuberculosis*, there was a notable decrease in glutathione peroxidase activity, which was markedly enhanced with treatment using neem extract. This indicates the potential of neem extract as a natural antioxidant and anti‐inflammatory agent in the fight against TB infection (Iqbal, Kumar, and Islam 2008). In summary, Neem bark, from *Azadirachta indica* A. Juss, is used for respiratory issues and is rich in antioxidants. Its compounds modulate genetic pathways, while flavonoids exhibit antibacterial effects.

Additionally, the edible plant garlic (*Allium sativum*) has piqued people's interest as a potential therapeutic. Sulphur compounds found in garlic include allicin, ajoene, diallyldisulfide, allylmethyltrisulfide, and others. The biological activities of these compounds encompass a range of effects, such as antibacterial, immunomodulatory, hypoglycemic, antioxidant, anti‐inflammatory, and cardiovascular actions (Viswanathan, Phadatare, and Mukne 2014). *A*. *sativum* demonstrates a potent mechanism of action against mycobacterial infections, exhibiting robust antituberculosis responses both in vitro and in vivo, targeting drug‐sensitive, MDR, and XDR strains. Allicin, a key component of garlic extract, demonstrates direct killing of mycobacteria. Additionally, allicin induces pro‐inflammatory cytokines in macrophages. In murine infection models, the administration of allicin extract triggers a strong protective Th1 immune response, resulting in a notable decrease in the load of mycobacteria. These results underscore the multifaceted nature of allicin/garlic extract, showcasing its dual role in both antibacterial action and modulation of the immune system. Notably, garlic extract also reverses the immune‐suppressive effects associated with frontline antituberculosis drugs (Dwivedi et al. 2019). The results from the genotoxicity assays indicate that *A*. *sativum* does not exhibit genotoxic effects. Additionally, the no‐observed‐adverse‐effect level (NOAEL) for GEO is determined to be higher than 50 mg/kg body weight per day when administered orally to mice for a duration of 28 days (Y.‐E. Lin et al. 2022). Additionally, in vitro investigations utilized the resazurin microtiter plate assay technique to assess the antimycobacterial potential of different garlic extracts, while the colony count method was employed to evaluate the efficacy of garlic oil. The antibacterial effectiveness of both the extracts and oil was determined using the zone of inhibition method. Extracts containing high levels of allicin and ajoene demonstrated noteworthy antimycobacterial properties in comparison to conventional medications (Viswanathan, Phadatare, and Mukne 2014). Additionally, in vitro screening using the resazurin microtiter plate assay (REMA) assessed the activity of individual isolates and garlic extract (GE) against *M*. *tuberculosis* H37Rv. GE exhibited greater antitubercular activity compared to isolates in REMA, possibly attributed to synergistic interactions among constituents of GE. Cytotoxicity assessments in RAW 264.7 mouse macrophage cells revealed a favorable selectivity index (> 10) for GE. Consequently, further assessment of the antitubercular activity of GE was conducted using a macrophage infection model, revealing concentration dependant activity in macrophages infected with H37Rv (Nair et al. 2017). In summary, garlic (*A*. *sativum*) contains sulfur compounds like allicin, offering diverse health benefits such as antibacterial, immunomodulatory, and cardiovascular effects. It shows promise against TB by directly killing mycobacteria and triggering a protective immune response in murine models. Additionally, garlic oil exhibited noteworthy antibacterial effects, particularly targeting MRSA, without adverse reactions (Viswanathan et al. 2014).

*N*. *sativa*, used in the treatment of COVID‐19, also plays a role in treating TB. Its constituent, thymoquinone (TQ), also exhibits antibacterial and antitubercular activities. Using RAW 264.7 cells, TQ lowered levels of intracellular *M*. *tuberculosis* H37Rv and XDR‐TB 72 h after infection. Additionally, it reduced H37Rv‐infected cells' levels of pro‐inflammatory chemicals including TNF‐α and IL‐6, as well as inducible nitric oxide synthase (iNOS), leading to a reduction in stress caused by the pathogen (Mahmud et al. 2017). In mice infected with *M*. *tuberculosis*, the higher dosage of TQ (50 mg/kg) lowered IL‐1β and IL‐4 levels, but Th1 and Th2 levels could not be balanced back to their original state (Olivianto et al. 2021). Ethanolic extracts from *N*. *sativa* seeds are also found to protect against renal damage that was caused by anti‐TB drugs in mice (Jaswal et al. 2022). This proves that *N*. *sativa* has much potential as complementary treatment or as treatment in *M*. *tuberculosis* infection. The LD50 values for *N*. *sativa* seed fixed oil varied widely in mice, ranging from 28.8 mL/kg to 3371 mg/kg, while high doses of aqueous, methanol, and chloroform extracts of *N*. *sativa* did not result in mortality. Subacute toxicity assessments revealed that even at doses as high as 6 g/kg, these extracts did not induce toxicity. Chronic toxicity investigations indicated slight toxicity at 2 mL/kg of *N*. *sativa* fixed oil. Cytotoxicity studies demonstrated that chloroform and petroleum ether extracts of *N*. *sativa* exhibited greater cytotoxic effects compared to other extracts. Overall, while *N*. *sativa* extracts showed varied toxicity levels, the seed fixed oil was found to have a wide range of LD50 values, and caution should be exercised, particularly with higher doses (Mashayekhi‐Sardoo, Rezaee, and Karimi 2020). In summary, *N*. *sativa*, known as black seed, contains TQ with antibacterial and antitubercular properties. However, caution is advised with higher doses of *N*. *sativa* extracts.

A comparative study of the effect of *W*. *somnifera* as an adjuvant to DOTS in patients with newly diagnosed sputum smear‐positive pulmonary TB has shown promising results. Ashwagandha (*W*. *somnifera* Linn.), a rejuvenating herb commonly used in the Indian subcontinent, has shown promise as a supportive treatment for TB due to its ability to boost the immune system. In a study, patients treated with Ashwagandha saw better results in clearing TB bacteria from their sputum, with 86.6% of them testing negative after 8 weeks compared to 76.6% in the placebo group. By 12 weeks, these patients also had significant increases in important immune cells (CD4 and CD8). Additionally, fewer patients in the Ashwagandha group had elevated liver enzymes and uric acid levels, indicators of better overall health, compared to the placebo group. Patients treated with Ashwagandha also reported greater improvements in their quality of life (R. Kumar et al. 2018). This herb contains active compounds like alkaloids and withanolides, which are believed to drive these beneficial effects. Other plants, such as *Chenopodium ambrosioides*, *Azadirachta indica*, and *Flourensia cernua*, also have similar active compounds, suggesting that they might offer comparable health benefits (Alzohairy 2016; Aranda‐Ledesma et al. 2022; Jesus et al. 2018). This study highlights the potential of bioactive compounds present as a valuable addition to traditional TB treatment, improving patient outcomes by enhancing immune function and overall well‐being. Table 4 shows a summary of all plants highlighted in this section.

**TABLE 4:** Medicinal plants with their respective active constituents and their properties to treat tuberculosis.

Plant	Active constituent	Method of delivery	Properties	References
*Piper longum* Linn	Piperlongumine (PL) and piperine	Oral	Inhibits inflammatory mediators and hinders the adherence of lymphocyte function‐associated antigen 1 to MH‐S cells following trehalose 6,6′‐dimycolate (TDM) activation, modulates the macrophage‐inducible Ca^2+^‐dependent lectin receptor (Mincle)‐Syk‐ERK signaling pathway.	Lu et al. 2021
*Flourensia cernua*	Flavonoid	—	Potent in vitro antioxidants, effectively mitigating the harmful effects of free radicals and display notable antibacterial effectiveness against *M*. *tuberculosis*.	Linares Braham, Palomo‐Ligas, and Nery‐Flores 2023
*Chenopodium ambrosioides*	Phenolics, flavonoids, saponins, ecdysteroids, and triterpenoids	Oral	Significant antimicrobial activity, inhibiting a diverse range of microorganisms, and exhibiting the lowest minimum inhibitory concentration (MIC) values against various bacteria.	Jesus et al. 2018; Kasali et al. 2022; Nguta et al. 2015
*Azadirachta indica* A. Juss	Nimbin, nimbidin, nimbolide, limonoids, quercetin, and ß‐sitosterol	Oral	Exhibited antitubercular activity, suppressed TNF‐α and MTB Ag85B proteins in a dose‐dependent manner, and decreases glutathione peroxidase activity. Take it orally with cautions.	Ashafa, Orekoya, and Yakubu 2012; Govindachari et al. 1998; Iqbal, Kumar, and Islam 2008; Nagasree et al. 2022
*Allium sativum*	Allicin, ajoene, diallyldisulfide, allylmethyltrisulfide	Oral	Induces pro‐inflammatory cytokines in macrophages and induction of a robust protective Th1 response, leading to a significant reduction in mycobacterial burden. Safe to use. Dose: > 50 mg/kg bw/day	Dwivedi et al. 2019; Y.‐E. Lin et al. 2022; Viswanathan, Phadatare, and Mukne 2014
*Nigella sativa*	Thymoquinone	Intramuscular Intraperitoneal	Lowered levels of intracellular *M*. *tuberculosis* H37Rv and XDR‐TB. Decreased levels of inducible nitric oxide synthase (iNOS) and pro‐inflammatory substances like TNF‐α and IL‐6 in cells infected with H37Rv, also lowers IL‐1β and IL‐4 levels. LD50 for *N*. *sativa* seed fixed oil in mice: 28.8 mL/kg to 3371 mg/kg. No mortality observed with 21 g/kg of *N*. *sativa*. Subacute toxicity: No toxicity up to 6 g/kg.	Mahmud et al. 2017; Mashayekhi‐Sardoo, Rezaee, and Karimi 2020; Olivianto et al. 2021

## 3 Challenges and Limitation of Using Medicinal Plants for Pulmonary Infections

The effectiveness and safety of medicinal plants are greatly influenced by the quality of the source material used in production. Numerous factors may affect the quality of the crops produced, including the time of the year it was harvested, region of agriculture, portions of the plant used, and the method of processing. The composition of the plant may vary as well, based on the climate and environmental circumstances in the area. Not only that, but it may also take a long time and a lot of plant material to isolate individual chemicals with the desired activity. Good agricultural and collection practices (GACP) have to be adhered to as well to ensure the quality of plants collected is standardized. Hence, quality assessments on raw materials for herbal medicines would be hard to conduct (Ekor 2014; Vaou et al. 2021). Moreover, difficulties in commercializing medicinal plants are rarely discussed in research, especially when it comes to the involvement of intermediaries, the boom–bust cycle, raw material readiness, and product quality (Astutik, Pretzsch, and Ndzifon Kimengsi 2019).

Absence of clinical studies that are well‐controlled and double‐blind to demonstrate the safety and efficacy of medicinal plants can be observed. This shows the lack of evidence of the effectiveness of herbal medicine, and considerable evidence suggests that they can lead to severe adverse reactions. The occurrence of adverse events from herbal medicine consumption can be attributed to various factors, including unintentional use of the wrong plant species, contaminations present such as toxic substances, excessive dosing, improper utilization by health‐care professionals or patients, and simultaneous use of herbal remedies with other medications (Ekor 2014; Vaou et al. 2021). Furthermore, the phenomenon of compound synergism in complex mixtures poses distinct challenges due to the limited technological advancements in examining several compounds operating on multiple biological targets (Vaou et al. 2021). Many manufacturers of herbal medicines lack sufficient knowledge or fail to emphasize the importance of taxonomic botany and documentation. Hence, further data would be required to fully understand the nature, frequency, and preventability of adverse reactions that can be caused by medicinal plants (Farah et al. 2000).

The limitations of plant drug delivery encompass various challenges including poor solubility, stability issues, short biological half‐life, rapid elimination, lack of targeted delivery, limited bioavailability, and potential toxicity (Conte et al. 2016). Many bioactive compounds derived from plants suffer from poor solubility in water, hindering their absorption and effectiveness (H. S. Rahman et al. 2020). Additionally, these compounds are often prone to degradation due to environmental factors (Nasim, Sandeep, and Mohanty 2022). Rapid metabolism and excretion further limit their efficacy, while traditional delivery methods may lack specificity, leading to off‐target effects and potential toxicity (Conte et al. 2016). For instance, the majority of biologically active components found in extracts, including flavonoids, tannins, and terpenoids, exhibit high water solubility. However, their absorption is often limited due to factors such as inability to penetrate cell lipid membranes, excessively large molecular size, or poor absorption rates. Consequently, this leads to reduced bioavailability and effectiveness of these compounds (Bonifácio et al. 2013).

## 4 Future Prospects and Conclusion

Natural products and their derivatives have been shown to have a major therapeutic effect on people's health in the modern era. Since bioactive components are unique to each plant and typically less hazardous than synthetic chemicals, each plant has its own medicinal properties (Elkordy et al. 2021). Hence, due to the factors including low cost compared to synthetic drugs, less adverse effects, and high public compliance, investigation in combining the medicinal plants with the synthetic therapeutic drug to achieve synergistic effect against the pulmonary infection is crucial for future medical field (Rahman et al. 2022; Wachtel‐Galor and Benzie 2011). Moreover, incorporation of medicinal plants into daily intake of food and drinks is also a promising avenue for addressing pulmonary infections (Rahman et al. 2022). For instances, medicinal herbs such as *A*. *Sativum*, *C*. *longa*, and *Solanum lycopersicum* that posses effects against pulmonary infections are commonly added into dishes not only to enhance the tastiness but also to boost immunity against lung infections. However, on plants that can be easily accessed by the public, safety and toxicity reports should be established so that overdosing or poisoning can be prevented.

Further research can be done on various plants and their constituents, as corporation of molecules that are hard to fuse in the mixture can be solved by the advancement in technology, including nanotechnology such as micelles, liposomes, gold NPs, and other nanocarriers (Dewi et al. 2022). More clinical studies can be done, especially on plants that have various biological properties on different infections, including *N*. *sativa*, *C*. *longa*, and *G*. *biloba*. In using an advanced drug delivery system, the compound's bioavailability would be increased as targeted delivery is achieved. Moreover, herbal medication constitutes a billion‐dollar industry, contributing significantly to the economy of certain countries, especially those conducive to growing specific medicinal plants due to their climate requirements.

In conclusion, bioactive compounds in plants are a vast field of knowledge that has a great potential to be fully utilized in the future. Various studies have proven the biological effect of various bioactive constituents in terms of pulmonary infection in both in vivo and in vitro, and some extending to clinical studies. Collaborative efforts between researchers, health‐care professionals, and policymakers are essential for integrating plant‐based medicine into mainstream health‐care practices. By leveraging the therapeutic potential of plant‐derived compounds and embracing innovative delivery systems, we can pave the way for more effective and sustainable approaches to managing pulmonary infections and improving public health globally.

## Author Contributions

**Joyce Siaw Syuen Ho:** conceptualization, writing – original draft. **Teh Li Ping:** conceptualization, writing – original draft. **Keshav Raj Paudel:** conceptualization, project administration, supervision, visualization. **Tammam El Sherkawi:** supervision, visualization. **Gabriele De Rubis:** supervision, visualization. **Stewart Yeung:** supervision, visualization. **Philip M. Hansbro:** conceptualization, supervision, visualization. **Brian Gregory George Oliver:** conceptualization, supervision, visualization. **Dinesh Kumar Chellappan:** conceptualization, supervision, visualization. **Keng Pei Sin:** conceptualization, project administration, supervision, visualization. **Kamal Dua:** conceptualization, project administration, supervision, visualization.

## Conflicts of Interest

The authors declare no conflicts of interest.

## Data Availability

No data has been used to compile this review.
